# Novel Extracellular Electron Transfer Channels in a Gram-Positive Thermophilic Bacterium

**DOI:** 10.3389/fmicb.2020.597818

**Published:** 2021-01-11

**Authors:** Sergey N. Gavrilov, Daria G. Zavarzina, Ivan M. Elizarov, Tamara V. Tikhonova, Natalia I. Dergousova, Vladimir O. Popov, Jonathan R. Lloyd, David Knight, Mohamed Y. El-Naggar, Sahand Pirbadian, Kar Man Leung, Frank T. Robb, Maksim V. Zakhartsev, Orianna Bretschger, Elizaveta A. Bonch-Osmolovskaya

**Affiliations:** ^1^Winogradsky Institute of Microbiology, FRC Biotechnology Russian Academy of Sciences, Moscow, Russia; ^2^Bach Institute of Biochemistry, Research Center of Biotechnology, Russian Academy of Sciences, Moscow, Russia; ^3^Kurchatov Complex NBICS-Technologies, National Research Center “Kurchatov Institute,” Moscow, Russia; ^4^Dalton Nuclear Institute, FSE Research Institutes, The University of Manchester, Manchester, United Kingdom; ^5^Faculty of Biology, Medicine and Health, The University of Manchester, Manchester, United Kingdom; ^6^University of Southern California, Los Angeles, CA, United States; ^7^School of Medicine, University of Maryland, Baltimore, Baltimore, MD, United States; ^8^Faculty of Biosciences, Norwegian University of Life Sciences, Ås, Norway; ^9^J. Craig Venter Institute, La Jolla, CA, United States; ^10^Faculty of Biology, Lomonosov Moscow State University, Moscow, Russia

**Keywords:** multiheme cytochromes, thermophilic prokaryotes, iron reduction, electrogenesis, biogenic magnetite crystals, Gram-positive bacteria

## Abstract

Biogenic transformation of Fe minerals, associated with extracellular electron transfer (EET), allows microorganisms to exploit high-potential refractory electron acceptors for energy generation. EET-capable thermophiles are dominated by hyperthermophilic archaea and Gram-positive bacteria. Information on their EET pathways is sparse. Here, we describe EET channels in the thermophilic Gram-positive bacterium *Carboxydothermus ferrireducens* that drive exoelectrogenesis and rapid conversion of amorphous mineral ferrihydrite to large magnetite crystals. Microscopic studies indicated biocontrolled formation of unusual formicary-like ultrastructure of the magnetite crystals and revealed active colonization of anodes in bioelectrochemical systems (BESs) by *C. ferrireducens*. The internal structure of micron-scale biogenic magnetite crystals is reported for the first time. Genome analysis and expression profiling revealed three constitutive *c*-type multiheme cytochromes involved in electron exchange with ferrihydrite or an anode, sharing insignificant homology with previously described EET-related cytochromes thus representing novel determinants of EET. Our studies identify these cytochromes as extracellular and reveal potentially novel mechanisms of cell-to-mineral interactions in thermal environments.

## Introduction

Extracellular electron transfer (EET) is a unique energy conservation mechanism of prokaryotic organisms that allows them to exploit a wide variety of redox-active minerals for energy generation and to maintain direct interspecies electron transfer in complex sedimentary milieus (Shi et al., 2016). Most studies of electron exchange between microbial cells and insoluble electron acceptors are based on the data obtained for diderm Gram-negative Fe(III) reducing microorganisms. Moreover, key genes and gene products enabling the EET to solid-phase Fe(III) minerals have been extensively characterized only in model species of mesophilic Proteobacteria belonging to the genera *Geobacter* and *Shewanella* (Shi et al., 2016).

Extracellular electron transfer processes drive important biogeochemical transformations in thermal sediments where insoluble Fe(III) compounds are abundant electron acceptors that can energize microbial life adapted to high temperature growth and survival (Kashefi et al., 2002). Thermophilic dissimilatory metal-reducers are dominated by monoderm prokaryotes (Firmicutes and hyperthermophilic Archaea), which represent many deep phylogenetic lineages (see Lusk, 2019 and the references therein). Their monolayer cell envelope obviates the need for soluble diffusible periplasmic electron shuttles and short-circuits their respiratory electron transport chains for access to such insoluble electron acceptors as Fe(III) or Mn(VI) oxides, ferruginous silicates, etc.

Current reviews highlight the key role of three groups of multiheme *c*-type cytochromes in EET in diderm bacteria (Shi et al., 2016; White et al., 2016). These include quinol-oxidizing cytochromes in the cytoplasmic membrane, electron-shuttling periplasmic cytochromes, and terminal respiratory oxidoreductases, transferring electrons directly to an insoluble acceptor on the cell surface (Xiong et al., 2006; Zhang et al., 2008). Two routes are generally proposed for the terminal electron transfer step (Shi et al., 2016; White et al., 2016). The first pathway is mediated by porin–cytochrome complexes linking intracellular electron shuttles with the cytochromes on the cell surface. Such complexes of Pcc- or Mtr-type are widespread among various iron-cycling prokaryotes (White et al., 2016), although it is still unclear whether these complexes are universal. The second pathway for the terminal step of EET is based on electrically conductive cell appendages (“nanowires”), that have been described as electron transferring type-IV pili, or “e-pili” (see Walker et al., 2018 for review), and later on argued to be stacks of cytochromes in *Geobacter* spp. (Filman et al., 2019; Wang et al., 2019) or cytochrome-functionalized membrane extensions in *Shewanella oneidensis* (Lovley and Malvankar, 2015; Subramanian et al., 2018). Cytochrome redox proteins and nanowires form multiple chimeric electron conduits in the genera *Geobacter* and *Shewanella* to support diverse electron transfer processes to different electron acceptors (Nevin et al., 2009; Coursolle and Gralnick, 2010; Kavanagh et al., 2016). EET determinants in the wide variety of prokaryotes with monolayer cell envelope are apparently highly diverse. However, limited information exists on them. Physiological mechanisms for Fe(III) respiration in the monoderm prokaryotes are cursorily reported for three Firmicutes, including two thermophiles (see White et al., 2016; Lusk, 2019 for reviews), and three hyperthermophilic archaea of the family *Archaeoglobaceae* (Manzella et al., 2015; Mardanov et al., 2015; Smith et al., 2015). The major role of multiheme *c*-type cytochromes in EET of all these organisms has been described, but specific electron transferring proteins involved in energy generation and their interactions with each other still remain poorly characterized. Recent reports on the electrically conductive archaellum from *Methanospirillum hungatei* (Walker et al., 2019) and multiheme *c*-type cytochromes involved in direct electron exchange between ANME archaea and sulfate-reducing bacteria (Krukenberg, 2018) support the notion that additional classes of electron transferring proteins remain to be discovered.

Significantly, EET pathways determine electrocatalytic activity of particular organisms and of whole microbial communities. Interaction with conductive minerals (such as magnetite) in natural environments and with charged electrodes in bioelectrochemical systems (BESs) allows prokaryotes to use these solid-state surfaces as electron acceptors or donors in the processes of microbial exoelectrogenesis or electrotrophy, respectively. Electrocatalytic activity has been well documented for a variety of pure, mixed, and enrichment prokaryotic cultures, as well as for natural sediments (Dopson et al., 2016; Nealson, 2017), but very little is known about the electrogenic activity in thermophiles. The information on their mechanisms of electrogenesis still remains limited to the detection of e-pili-like appendages in *Thermincola ferriacetica* biofilms (Lusk, 2019), heterologous expression of e-pili genes from *Calditerrivibrio nitroreducens* (Walker et al., 2018), and biochemical characterization of recombinant EET-related multiheme cytochromes OcwA and TherjR_0333 from “*Thermincola potens*” (Costa et al., 2015, 2019). OcwA, associated with the cell surface and possessing iron-reducing activity, revealed structural similarity to octaheme cytochromes involved in nitrate and sulfate reduction. Considering this fact and wide potential window of the cytochrome’s activity, the authors proposed its multifunctional role in the respiratory metabolism and evolutionary closeness to the octahemes but not the known terminal reductases involved in EET (Costa et al., 2019).

Here we report exoelectrogenic and magnetite-crystallizing activity of the Gram-positive dissimilatory Fe(III)-reducing bacterium *Carboxydothermus ferrireducens*, and identify the determinants of these processes at both genomic and protein levels.

*Carboxydothermus ferrireducens* is an anaerobic, thermophilic bacterium isolated from a hot spring at Yellowstone National Park (Slobodkin et al., 1997). The organism belongs to the clostridial family *Thermoanaerobacteraceae* according to rRNA-based taxonomy or together with its closest relatives comprises a separate deep lineage “*Carboxydothermales*” in the class “*Desulfotomaculia*” proposed by genome-based phylogeny (Parks et al., 2018). *C. ferrireducens* couples the oxidation of organic and inorganic electron donors to the reduction of a variety of electron acceptors, including soluble and insoluble Fe(III) forms and sparingly soluble U(VI) minerals, as well as bicarbonate (in homoacetogenic growth driven by Wood-Ljungdahl pathway). The organism has a typical Gram-positive cell wall, enclosed by an *S*-layer. Our previous studies revealed that direct cell-to-mineral contact is the major physiological strategy for ferrihydrite reduction in the organism, and this strategy is promoted by cell surface-associated *c*-type cytochromes (Slobodkin et al., 1997, 2006; Gavrilov et al., 2012; Toshchakov et al., 2018). The organism does not produce any soluble electron shuttles or iron chelators which could serve as the mediators of EET (Gavrilov et al., 2012). In the current study, we describe several novel secreted multiheme cytochromes, fairly distantly related to previously reported determinants of EET, and provide evidence for their metabolic significance and differential involvement in electron transfer chains (ETCs) to different insoluble electron acceptors, Fe(III) oxide ferrihydrite, and the anodes of BESs.

## Materials and Methods

### Cultivation Conditions

*Carboxydothermus ferrireducens* strain DSM 11255^*T*^ was sustained in an anaerobic bicarbonate-buffered medium with poorly crystalline Fe(III) oxide ferrihydrite containing 90 mM Fe(III) (Slobodkin et al., 1997). For the experiments with different electron acceptors, the same type media lacking ferrihydrite and containing either ferric citrate [20 mM Fe(III)], sodium fumarate (20 mM) (Gavrilov et al., 2012), or none of these electron acceptors but instead a stainless steel or graphite electrode (see below) were used. Glycerol (33 mM) was used as an electron donor and a carbon source and yeast extract (100 mg/L)—as a growth factor throughout all the cultivation experiments. Cultures were maintained in Hungate tubes at 65°C, biomass for proteomic profiling and cell fractionation was obtained in the same conditions in 100 mL and 1 L serum bottles, respectively, the bottles were sealed with butyl rubber stoppers and screw caps. Current production by *C. ferrireducens* was studied using dual-chamber BESs, total volume 300 mL (Glass Reactor Set 948113, Adams & Chittenden Scientific Glass, Berkeley, CA, United States), as described previously (Bretschger et al., 2007). In all the cultivation vessels, except BESs, 70% of the vessel volume was filled with liquid medium, and 30%—with extra pure CO_2_ gas. In the BESs, anodic chambers were completely filled with the liquid medium lacking any electron acceptors prior to inoculation.

### Inoculation Techniques

All the transfers were made anaerobically with syringes and needles, 1% inoculum volume was used throughout. For proteomic profiling across different electron acceptors, the cultures were two times subsequently transferred from Hungate tubes with ferrihydrite medium to the tubes containing the media with corresponding electron acceptors (20 mM fumarate or ferric citrate) and third transfers from Hungate tubes to 100 mL serum bottles were used for biomass harvesting. BESs initially contained the medium without electron acceptors and were inoculated with ferrihydrite-grown cultures with residual magnetite. The inocula were taken with syringes and needles after preliminary separation of cells from the bulk of magnetite with a neodymium hand magnet attached to the Hungate tube. The total Fe content of the medium in BESs after inoculation comprised ca. 600 μM [200 μM Fe(II) and 400 μM Fe(III)].

### Determination of Growth and Fe(III) Reduction Kinetics

Growth with soluble electron acceptors was determined by direct cell counting under a CX41 phase-contrast microscope (Olympus). For counting the cells growing with ferrihydrite, subsamples of the cultures were diluted with the mixture of ammonium oxalate and oxalic acid to dissolve iron-containing minerals as previously described (Lovley and Phillips, 1988). Growth of electrogenic cells was estimated by visual observations of biofilms appearance on BES’s anodes as well as by direct counting of planktonic cells in the anolyte. Iron(III) reduction kinetics was traced by determination of HCl-extractable Fe(II) species in culture subsamples with ferrozine as previously described (Gavrilov et al., 2012).

### SEM and FIB Studies

For scanning electron microscopy (SEM) studies, ferrihydrite-grown cultures in stationary growth phase were used, and subsamples were taken anaerobically with syringes and needles. These subsamples were fixed in 2.5% glutaraldehyde before being subjected to dehydration series (25, 50, 75, 90, and 100% v:v ethanol, 15 min each treatment) and critical point drying (Autosamdri 815 critical point drier, Tousimis Inc., Rockville, MD, United States). The samples were then imaged at 10 keV using a JEOL JSM 7001F low vacuum field emission SEM. Focused ion beam (FIB) milling and associated SEM imaging of magnetite crystals were performed using a JEOL JIB-4500 FIB/SEM.

### Electrocatalytic Activity Determination

Two bioelectrochemical reactors were used: one operated on microbial fuel cell (MFC) mode, and another one operated on potentiostatic mode. All the BES parts (except membranes and reference electrodes) were autoclaved at 121°C for 20 min and further assembled and filled with pre-sterilized culture medium aseptically in UV-irradiated anaerobic glove box (98% N_2_, 2% H_2_). Nafion 242 proton-exchange membranes (DuPont) and Ag/AgCl reference electrodes (+200 mV vs. SHE, RE-5B, BASi, IN, United States) were UV-irradiated for 20 min before BESs assembling. In MFC reactors, graphite felt electrodes (ca. 80 cm^2^ surface area; GF-S6-06; Electrolytica) bonded to platinum wires (0.3 mm; Alfa-Aesar) were used, while potentiostatic mode reactors were assembled with meshy stainless steel electrodes (240 mesh, 80 cm^2^ surface area) bonded to 0.4 mm titanium wires (both from Joyetech, China) to simplify shearing of biofilms and extracellular proteins from anodes. The culture medium lacking external electron acceptors was used in anodic chambers of both BESs types. Cathodic chambers in potentiostatic mode BESs were filled with the same sterile culture medium. In MFCs, cathodic chambers were filled with potassium ferricyanide solution. MFCs were incubated for 3 days with open circuit in the bicarbonate-buffered medium to pregrow biofilms on graphite surface, and were further operated with a 200 Ω resistor load. Before closing the circuit, samples of the pregrown *C. ferrireducens* biofilms were collected anaerobically, stained with SYBR Green fluorescent DNA stain (Sigma–Aldrich) according to the manufacturer’s recommendations, and imaged using a Leica confocal laser microscope. The potentiostat mode BESs were turned on right after inoculation with anodes poised at −60 mV vs. SHE. Anode potential was controlled using the reference electrodes with IPC-micro potentiostats (Cronas, Russia). Three different potentiostatic BESs were inoculated from the same source culture and started simultaneously. Potentiostatic BESs were operated continuously for 9 days with phase-contrast microscopic control of cell growth (see above) until appearance of transparent lysing planktonic cells in the anodic chamber that corresponded to the formation of visible biofilms on the anodic surface. After the end of incubation, subsamples of anodes with biofilms were imaged using a Leica confocal laser microscope as described above. The biofilms were used to obtain biomass of electrogenic cells for proteomic studies. The biofilms were sheared from stainless steel anodes by rigorous shaking in the culture broth, and the cells were further separated by centrifugation at 16,000 *g* 15 min.

### Genome Analysis

The closed genome sequence of *C. ferrireducens* was obtained at the DOE Joint Genome Institute by Pacbio sequencing. Screening of the *C. ferrireducens* genome for multiheme cytochromes and their sequence analysis was performed as previously described (Toshchakov et al., 2018) using reported cytochromes, involved in EET in *Geobacter sulfurreducens* (Butler et al., 2010; Aklujkar et al., 2013), *S. oneidensis* (Coursolle and Gralnick, 2010; Schicklberger et al., 2013), and “*T. potens*” (Carlson et al., 2012), as queries, heme-binding motifs were predicted as previously described (Mardanov et al., 2015).

### Fractionation of Ferrihydrite-Grown Cultures

Cells were cultivated in 1 L bottles and separated from magnetite, produced by the end of logarithmic growth, by rigorous hand shaking of culture bottles for 5 min and further centrifugation of the broth at 1000 *g* 5 min. Hereafter, all the centrifugation steps were performed at +4°C. The resulting supernatant with magnetite-free cell suspension was further fractionated, and the mineral pellet was washed three times with PBS-Tween buffer (pH 7.5) and finally, with 0.1% SDS. The resulting SDS outwash of magnetite was separated by centrifugation (16,000 *g*, 15 min), concentrated 20 times using Microcon YM-3 centrifugal filter devices (3 kDa cut-off membrane, Millipore Co.) and used for further MS-identification of the proteins stuck to magnetite.

The magnetite-free cell suspension was halved. One portion was used for shearing the cell surface proteins according to the previously described procedure (Mehta et al., 2005). The resulting PBS-buffered protein suspension was centrifuged for 30 min at 16,000 *g* to remove debris, and the obtained supernatant was used for identification of cell surface-associated proteins. Whole cells from another portion of magnetite-free cell suspension were pelleted at 16,000 *g* for 15 min, resuspended at pH 8.0 in 50 mM Tris-HCl buffer, then ultrasonicated, and further centrifuged at 100,000 *g* for 40 min in order to separate soluble and insoluble proteins.

### Identification of Target Proteins in Subcellular Fractions

All protein samples from subcellular fractions were first routinely analyzed with SDS-PAGE and the gels were stained with Coomassie brilliant blue R-250 for proteins. For identification of the proteins associated with the cell surface or magnetite crystals, all the protein bands were prepared for further LC-MS/MS analysis using nanoAcquity LC (Waters) coupled to a LTQ Velos Pro (Thermo Fisher Scientific) mass spectrometer. To identify heme-containing proteins in subcellular fractions and cell extracts, parallel preparations with the same protein load were run on the gels and separately stained with Coomassie for proteins or benzidine for hemes according to Francis and Becker (1984). Coomassie-stained protein bands corresponding to heme bands were excised from gels and dehydrated using acetonitrile followed by vacuum centrifugation. Dried gel pieces were reduced with 10 mM dithiothreitol and alkylated with 55 mM iodoacetamide. Gel pieces were then washed alternately with 25 mM ammonium bicarbonate followed by acetonitrile. This was repeated, and the gel pieces were dried by vacuum centrifugation. Samples were digested with trypsin overnight at 37°C. Peptide mixtures of tryptic digests were separated by liquid chromatography with a gradient from 92% solution A [0.1% formic acid (FA) in water] and 8% solution B (0.1% FA in acetonitrile) to 67% A and 33% B, in 44 min at 300 nL min^–1^, using a 0.25 × 75 mm, 1.7 μm, ethylene bridged hybrid C18 analytical column (Waters). Peptides were selected for fragmentation automatically by data-dependent analysis and subjected to MALDI-TOF MS on an UltrafleXtreme mass-spectrometer (Bruker). Mass spectra were analyzed as follows. The peak lists identified from MS spectra were subjected to the Peptide Mass Fingerprint (PMF) search at MASCOT Server v2.6 against *C. ferrireducens* amino acid FASTA dataset (2554 protein coding sequences; 739,902 residues, IMG database genome ID 2844895905). The common contaminants database was included into the search. The following parameters were employed for the PMF search: protease—trypsin; max missed cleavages—2; fixed modifications—carbamidomethyl; variable modifications—oxidation; mass values—monoisotopic; protein mass—unrestricted; peptide mass tolerance ± 100 ppm; peptide charge state 1^+^; number of queries 103. The peak lists identified from MS/MS spectra were subjected to the MS/MS Ion search against the same FASTA database with the same parameters but peptide mass tolerance adjusted to ± 1.2 Da; fragment mass tolerance ± 100 ppm; instrument type—MALDI-TOF-TOF. Protein scores were calculated as −10⋅log(P), where P is the probability that the observed match is a random event. The scores greater than 47 were regarded significant (*p*-value < 0.05).

### Shotgun Proteomic Analysis

For proteomic analysis, biomass was harvested from 100 mL cultures by centrifugation at 16,000 *g* for 15 min. Cell lysis, reduction, alkylation, and digestion of the proteins were performed as follows. Sodium deoxycholate (SDC) lysis, reduction, and alkylation buffer (pH 8.5) containing 100 mM TRIS, 1% (w/v) SDC, 10 mM TRIS(2-carboxyethyl)phosphine, and 40 mM 2-chloroacetamide was added to a biomass sample. The sample was sonicated and boiled for 10 min, protein concentration in the sample was routinely determined by Bradford assay, and equal volume of trypsin solution (pH 8.5) in 100 mM TRIS was added in a 1:100 (w/w) ratio. After overnight digestion at 37^°^C, peptides were acidified by 1% trifluoroacetic acid (TFA) for styrenedivinylbenzene reverse-phase sulfonate (SDB-RPS)-binding, and 20 μg was loaded on two 14-gauge StageTip plugs. Equal volume of ethyl acetate was added, and the StageTips were centrifuged at 300 *g*. After washing the StageTips with a 100 μL of 1% TFA/ethyl acetate mixture and 100 μL of 0.2% TFA, peptides were eluted by 60 μL acetonitrile/ammonia (80/5%, v/v) mixture. The collected material was vacuum-dried and stored at −80^°^C. Before analysis, peptides were dissolved in 2% acetonitrile/0.1% TFA buffer and sonicated for 2 min. LC-MS/MS analysis was commercially performed in the core facility center “Bioorganic” (IBCh RAS, Moscow, Russia) using the Q Exactive HF benchtop Orbitrap mass spectrometer coupled to the Ultimate 3000 Nano LC System via a nanoelectrospray source (all from Thermo Fisher Scientific). Dissolved peptides were analyzed using the HPLC system configured in a trap-elute mode. Approximately 1 μg of tryptic peptide digests was loaded on an Acclaim PepMap 100 (100 μm × 2 cm) trap column and separated on an Acclaim PepMap 100 (75 μm × 50 cm) column (both from Thermo Fisher Scientific). Peptides were loaded in solvent A (0.2% FA) and eluted at a flow rate of 350 nL min^–1^ with a multistep linear gradient of solvent B (0.1% FA, 80% acetonitrile): 4–6% B for 5 min; 6–28% B for 91 min; 28–45% B for 20 min; 45–99% B for 4 min; 99% B for 7 min; 99–4% B for 1 min. After each gradient, the column was washed with 96% buffer B for 9 min. Column was kept at 40°C. Peptides were analyzed on a mass spectrometer with one full scan (375–1,400 *m*/*z*, *R* = 60,000 at 200 *m*/*z*) at a target of 3e6 ions and max ion fill time 30 ms, followed by up to 15 data-dependent MS/MS scans with higher-energy collisional dissociation (target 1e5 ions, max ion fill time 50 ms, isolation window 1.2 *m/z*, normalized collision energy 28%, underfill ratio 2%), detected in the Orbitrap (*R* = 15,000 at fixed first mass 100 *m*/*z*). Other settings: charge exclusion—unassigned, 1, >6; peptide match—preferred; exclude isotopes—on; dynamic exclusion—30 s was enabled.

Label-free protein quantification was made by MaxQuant software version 1.5.6.5 using *C. ferrireducens* amino acid FASTA dataset (see above) and a common contaminants database by the Andromeda search engine, with cysteine carbamidomethylation as a fixed modification and protein N-terminal acetylation and methionine oxidations as variable modifications. The false discovery rate (FDR) was set to 0.01 for both proteins and peptides with a minimum length of seven amino acids. Peptide identification was performed with an allowed initial precursor mass deviation up to 20 ppm and an allowed fragment mass deviation of 20 ppm. Downstream bioinformatics analysis was performed with Perseus.1.5.5.1. For Student’s *t*-test, missing values were imputed with a width of 0.3 and a downshift of 1.8 over the total matrix. Two sample tests were performed in Perseus with s0 set to 0. Quantification was performed with a minimum ratio count of 1. To quantify proteins in each sample, the iBAQ algorithm, implemented into MaxQuant software, was used (Schwanhäusser et al., 2011). Normalization of each protein’s iBAQ value to the sum of all iBAQ values generated a relative iBAQ (riBAQ) values corresponding to the mole percentage of each protein in the sample, taking the whole set of proteins in it as 100% (Shin et al., 2013).

### Statistical Analysis of Proteomic Data

Two separate two-way ANOVA tests with multiple comparisons were performed using Graphpad Prism 8 software^1^ (i) electron acceptors (column factor) as the main source of variation vs. five groups of proteins (row factor) determining housekeeping, respiratory, and EET processes in *C. ferrireducens* and (ii) electron acceptors (column factor) vs. individual multiheme cytochromes from the group V (row factor). Descriptions of the proteins and their groups are given in the main text below and in the legends to Figures 5, 6. The group statistics (averages, standard deviations, and *n* values) has been calculated for each of five protein groups, or each of individual cytochrome proteins, in order to investigate their contribution into overall variance. Each two-way ANOVA consisted of two steps: (i) ordinary two-way ANOVA tests with *P*-value cut-off < 0.05 (Supplementary Tables S1, S2) and (ii) ANOVA tests corrected for multiple comparisons controlling FDR at Q = 0.05 (two-stage linear step-up procedure of Benjamini, Krieger, and Yekuteli with individual variances computed for each comparison). Pairwise comparisons of representation of each protein group at each culture conditions were calculated (number of families 1, number of comparisons per families 190, Q = 0.05) (Supplementary Table S3), and the same was done for each of individual multiheme cytochromes (number of families 10, number of comparisons per families 6, Q = 0.05) (Supplementary Table S4).

### Phylogenetic Analysis

Cytochrome *c* protein sequences related to the proteins OmhA, SmhA, SmhB, and SmhC were retrieved from non-redundant NCBI protein database on March 2020, using the BLASTp algorithm (Altschul et al., 1990) with default parameters except the following: model sequences (XM/XP) were excluded, automatic adjustment for short input sequences was restricted, low complexity regions filter was on, max target sequences parameter was adjusted to 10,000, the expectation threshold to 1, and the gap costs to existence 11 and extension 2. The retrieved selections of sequences were manually curated. Considered meaningful were the hits with Ev < 0.001, query coverage ≥ 50%, and identity ≥ 20%, as well as the hits with query coverage between 50 and 20% having Ev ≥ 10^–5^ or a percentage identity ≥ 70% at an Ev ≥ 0.001. These criteria were derived from several comprehensive systematic studies of multiheme cytochromes diversity (Butler et al., 2010; Sharma et al., 2010; Aklujkar et al., 2013; Shi et al., 2014). The resulting selections were further checked for ‘‘improper’’ hits lacking conservative multiheme domains or having no matches with the queries within such domains. These hits were excluded from further analysis. The obtained sets of sequences were then consecutively filtered through 0.95 and 0.80 filters using CD-hit utility^2^ to decrease redundancy. For SmhA hits, an additional third 0.60 filter was applied due to the large number of similar blast hits. The selections were then aligned by MAFFT 7 with default parameters using iterative refinement method FFT-NS-i (Katoh et al., 2019). Three final alignments (for SmhA, SmhB, and OmhA-SmhC hits) were subjected to a Bayesian inference of phylogeny using the BEAST package (BEAUti v2.6.2, BEAST v2.6.2, TreeAnnotator v2.6.0, FigTree v.1.4.4) (Bouckaert et al., 2019). Tree likelihoods (ignoring ambiguities) were determined for the alignments by creating a Monte-Carlo Markov Chain (10,000,000 generations) in three independent runs. The searches were conducted assuming an equal or a gamma distribution of rates across sites, sampling every 1000th generation and using the WAG empirical amino acid substitution model (Whelan and Goldman, 2001). In each case, the resulting 10,000 trees (omitting the first 400 trees as burn-in) were used to construct unrooted phylogenetic consensus trees. For the analysis of the trees, taxonomic information on the source organisms was retrieved from NCBI Taxonomy database, and heme numbers of the proteins were determined by screening the sequences from the alignments for heme *c*-binding motifs using CLC Genomics Workbench 9.0.

### Data Availability

All data for understanding and assessing the conclusions of this research are available in the main text, SI Appendix, DOE’s JGI Integrated Microbial Genomes and Microbiomes database^3^, ProteomeXchange Consortium^4^ via the PRIDE partner repository with identifier PXD018523 (Perez-Riverol et al., 2019).

## Results

### Growth of *C. ferrireducens* on Ferrihydrite. Cell-to-Mineral Contacts Control Nucleation of Magnetite Crystals

**FIGURE 1:**
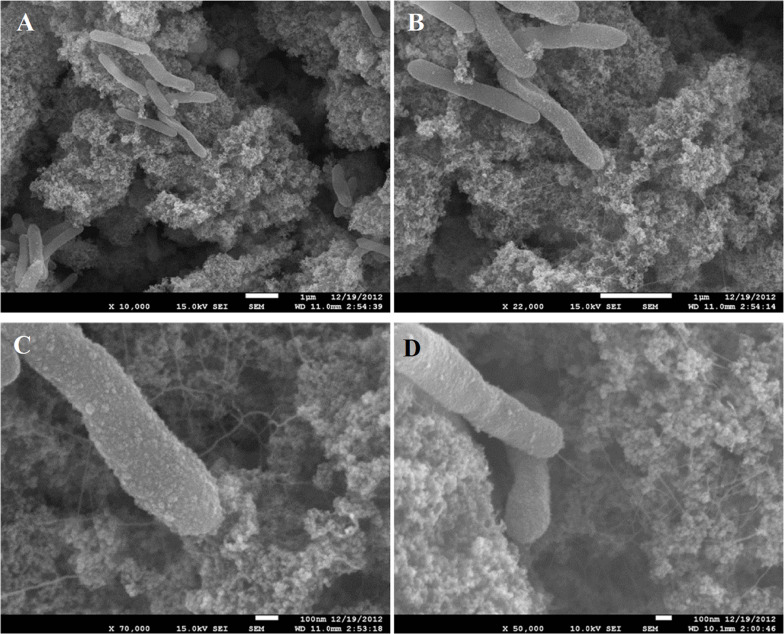
SEM images of small clusters of *C. ferrireducens* cells grown with ferrihydrite as sole electron acceptor. Surrounding material—nanoparticulate magnetite. **(A–C)** consecutive magnification of the same microscopic field. **(D)** Another microscopic field with a cell connected with the bulk of the mineral by a single appendage.

Growth of *C. ferrireducens* with ferrihydrite as the only electron acceptor is accompanied by the formation of biogenic cell-magnetite conglomerates (Gavrilov et al., 2012). Confocal fluorescent microscopy revealed irregular colonization of the mineral surface in two different ways—with small clusters of several cells and with much larger conglomerates of ca. 10 μm in diameter (Supplementary Figure S1). SEM showed that the small cell clusters are aggregated with nanosized mineral particles, retaining the morphology of ferrihydrite. The cells in these clusters contact the bulk of the mineral phase with a network of tiny filaments, resembling pili (Figures 1A–D). We have previously reported the production of such cell appendages in *C. ferrireducens* in response to the presence of ferrihydrite in the medium (Gavrilov et al., 2012). Analysis of the appendages with scanned conductance (SCM, Bockrath et al., 2002) and conducting probe atomic force (CP-AFM, El-Naggar et al., 2010) microscopy in this study did not detect their electrical conductivity (Supplementary Figure S2).

**FIGURE 2:**
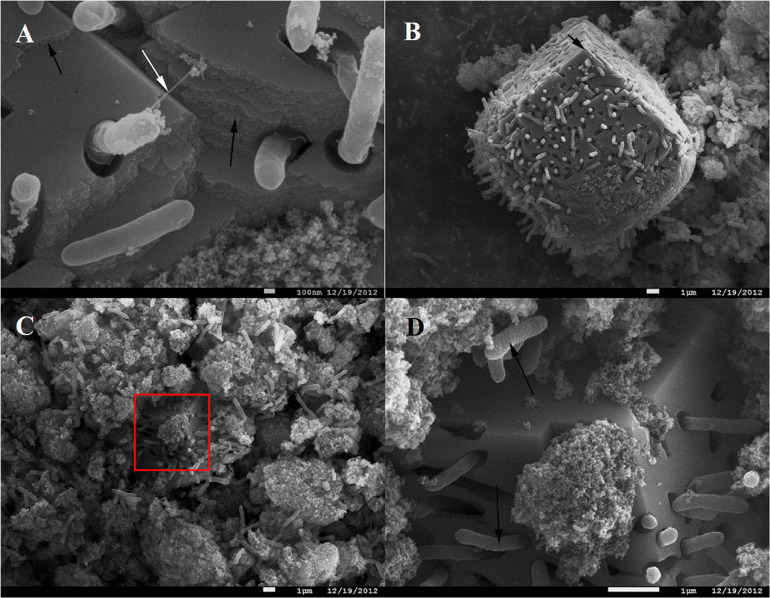
SEM images of *C. ferrireducens* cell colonies associated with macroscopic magnetite crystals in the late logarithmic phase of growth with ferrihydrite. **(A)** Non-piliated and piliated cells on the surface of a large magnetite crystal. **(B)** Overview of a large magnetite crystal. The *white arrow* on **(A)** indicates a singular cell appendage connecting the cell entrapped into magnetite crystal with the mineral surface, almost all the other cells associated with the crystal are non-piliated. *Black arrows* on **(A,B)** indicate incremental growth steps and facets of macroscopic magnetite crystals. **(C)** Different cell aggregates—inside macroscopic magnetite crystals and on the surface of nanoparticulate mineral phase. The red box on **(C)** indicates the area magnified at **(D)**. **(D)** Cells and cell-like cavities on the surface of a large (micron-scale) magnetite crystal. *Black arrows* on **(D)** point out different surfaces of the cells contacting the nanoparticulate mineral phase and the cells entrapped into large magnetite crystals (rough and smooth cell surfaces, respectively).

In contrast to the small clusters of “piliated” cells, big cell conglomerates in *C. ferrireducens’* cultures are confined within unusually large (≥10 μm) fine-grained sintered magnetite crystals (Figure 2). Such crystals comprise the majority of the mineral phase at the end of logarithmic growth of the culture. No further increase in their number or dimensions was detected by SEM after cell lysis. The majority of *C. ferrireducens* cells, associated with large magnetite crystals, lack extracellular filaments (Figure 2A). The large crystals have pronounced growth steps and well developed facets (Figures 2B,C) but lack ideal crystallographic form, which is consistent with rapid crystallization that has been abruptly stopped. The cells associated with the large magnetite crystals outnumber the cells bound to the surrounding mineral nanoparticles (Figures 2B–D and Supplementary Figure S1). Considering this fact, we propose that actively growing microcolonies of *C. ferrireducens* can act as centers of magnetite crystallization. Crystal growth takes place during active cell division and Fe(III) reduction with further Fe(II) mineralization on the cell surfaces. This may lead to the formation of macroscopic microbe-to-mineral conglomerates, ultimately limited by the cell lysis. Interestingly, FIB-milling of the large magnetite crystals revealed cavities matching the shape of the cells. Nanocrystalline filamentous structures, which can pick the cells, were not observed inside the cell-like cavities (Supplementary Figure S3), thus indicating the crystallization process to be somehow controlled by the cells entrapped into magnetite.

### Electrogenic Activity of *C. ferrireducens*

**FIGURE 3:**
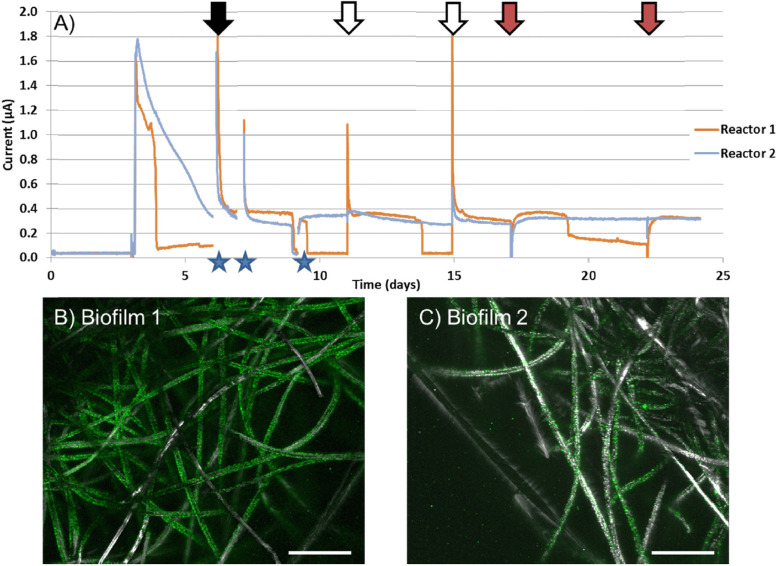
**(A)** Current vs. time of duplicate MFC reactors with *C. ferrireducens* biofilms pregrown at open circuit anodes with glycerol as the electron donor and bicarbonate as the electron acceptor. **(B)** Confocal image of pregrown *C. ferrireducens* biofilm used for reactor 1 (with more biomass pregrown), stained with SYBR Green fluorescent DNA stain. **(C)** Confocal image of pregrown *C. ferrireducens* biofilm used for reactor 2 (with less biomass pregrown). *Stars* indicate time periods where the circuit was opened to measure anodic and cathodic open-circuit potentials. *Black arrows* indicate full media exchanges. *White arrows* indicate additions of glycerol and yeast extract only. *Red arrows* indicate addition of glycerol, yeast extract, and ferrihydrite simultaneously. Scale bars on **(B,C)** are 200 μm.

Considering active colonization of magnetite by *C. ferrireducens*, which implies direct electron transfer from microbial cells to the conductive mineral, we decided to evaluate electrogenic activity of the organism in two high-temperature BESs operated in MFC or potentiostatic mode. Current generation was first observed in duplicate MFC-type reactors (Figure 3A) with biofilms of *C. ferrireducens* pregrown at open circuit anodes (Figures 3B,C) as homoacetogens using bicarbonate of the culture medium as the main electron acceptor available. After closing the circuit of MFC reactors, their anodes have become available for the cells as the electron acceptors, which led to current production. The higher biomass coverage on the anode in reactor 1 (Figure 3B) caused faster electron donor consumption rates as is indicated in Figure 3A. Biomass coverage on the graphite felt electrode was lower for reactor 2 (Figure 3C), and therefore longer intervals between feedings (Figure 3A, *blue lines*). Strong positive current response was observed upon addition of the electron donor glycerol to the medium or complete medium exchange (Figure 3A). Interestingly, when ferrihydrite was added to the reactors, an immediate negative current response was observed followed by a rapid recovery back to previous current levels (Figure 3A). These data likely indicate increased scavenging of electrons, derived from glycerol oxidation, in the presence of an extra electron acceptor (Fe^3+^ mineral) in the cultures.

In potentiostatic BESs with stainless steel anodes poised at -60 mV vs. SHE, no biofilms were pregrown at open circuit electrodes. Instead, the anodic chamber was inoculated with *C. ferrireducens* culture grown with ferrihydrite and containing magnetite nanoparticles (as those surrounding the cells in Figures 1C,D). Initial insoluble Fe content of these BESs comprised 600 μM. Within 9 days of incubation, clearly visible thin transparent biofilms with particulate magnetite inclusions appeared on the anodes (Supplementary Figures S4A–D) and have steadily generated 55–68 mA/m^2^ current since the second day of incubation (Supplementary Figure S4E). The current density appeared to be much higher than observed with biofilms pregrown on graphite anodes (0.04–0.08 mA/m^2^, Figure 3A).

### Genome Analysis

Potential determinants of EET to Fe(III) oxide or anode were annotated in the complete genome sequence of *C. ferrireducens*, revealing a total of 19 genes encoding multiheme *c*-type cytochromes with 4–22 predicted heme-binding motifs per protein sequence. Eighteen multihemes were predicted to be secreted proteins or localized on the cell surface. Thus, any of them could be involved in the terminal step of EET. The genes encoding secreted multihemes Ga0395992_02_129131_130396 and Ga0395992_02_130419_130832 comprise a typical cluster of NrfAH dissimilatory nitrite reductase complex and were excluded from further analysis. Three of the *C. ferrireducens’* multihemes (Ga0395992_01_217646_219709, Ga0395992_01_220048_222057, Ga0395992_01_222075_224618) are encoded in a large cluster containing the complete gene set for type-IV pili assembly (listed in Table 1 and Supplementary Table S5). The structural pilin PilA, encoded in this “pilin-cytochrome” cluster, was reported to lack homology with conductive e-pilins (Holmes et al., 2016). This gene cluster also encodes a “PilA–C” protein, with a C-terminus of similar topology to long type-IVa (poorly conductive) pilins and an N-terminus that is similar to porin-like proteins (Holmes et al., 2016). No genes related to the known determinants of Pcc- or Mtr-type porin-cytochrome complexes were identified in *C. ferrireducens*. Outside the “pilin-cytochrome” and *nrfAH* gene clusters, all the other cytochrome-encoding genes are randomly distributed throughout the *C. ferrireducens* genome. Among those, five proteins share homology with previously characterized parts of EET chains in iron-reducing bacteria *G. sulfurreducens*, *S. oneidensis*, and “*T. potens*” (Shi et al., 2016). Cytochrome Ga0395992_01_182949_184436 is homologous to OmcC outer surface cytochrome of the Pcc-type complex in the Gram-negative *G. sulfurreducens* (Aklujkar et al., 2013), Ga0395992_02_30996_31874 and Ga0395992_02_151260_153788 are homologous to MtrA/MtrD periplasmic cytochromes of the Mtr-type complex in the Gram-negative *S. oneidensis*, Ga0395992_01_647313_648317 is homologous to CymA quinol-oxidizing cytochrome of *S. oneidensis*, while Ga0395992_02_29558_30178 is homologous to periplasmic multiheme DmsE of *S. oneidensis*, as well as to putative soluble secreted cytochromes TherJR_0333, TherJR_1117 of “*T. potens*,” and Ga0395992_02_112574_114619 shares equally weak homology (23–30% identities) with three previously reported multihemes (Supplementary Table S5)—periplasmic cytochrome MtrA of *S. oneidensis*, OmcB cell surface-associated cytochrome of *G. sulfurreducens* (Butler et al., 2010), and OcwA outer surface cytochrome of the Gram-positive “*T. potens*” (Costa et al., 2019). Sequence analysis of Ga0395992_02_112574_114619 with CW-PRED service indicates its putative localization on the outer surface of the cell. Another *C. ferrireducens* multiheme, Ga0395992_03_307983_310529, shares weak homology with predicted cell wall-linked multiheme TherJR_1122 of “*T. potens*” (Carlson et al., 2012). Several multihemes of *C. ferrireducens*, including putative secreted octaheme Ga0395992_02_137389_139104, appeared to lack homologs among reported determinants of EET. Two multihemes of *C. ferrireducens* (Ga0395992_02_135863_137242 and Ga0395992_01_684739_686151) possess conserved domains of membrane anchor proteins of CISM family enzymes (Rothery et al., 2008), and thus might serve as quinol-oxidizing units transferring electrons from the quinol pool to terminal oxidoreductases. Alternative candidate quinol-oxidizing protein is Ga0395992_01_187284_188528, possessing conserved domain organization and number of hemes similar to those of ActA subunit of the respiratory alternative complex III (Refojo et al., 2013).

**TABLE 1:** Putative EET-related multiheme cytochromes of *C. ferrireducens* and the summary of their best blast hits among the components of EET pathways in *Shewanella oneidensis* MR-1, *Geobacter sulfurreducens* PCA, and “*Thermincola potens*.”

**Protein symbol^1^**	**Locus tag**	**Predicted number of heme-binding motifs**	**Predicted^2^ localization**	**Best blast hit**	**Min E-value**	**Max% Ident.**	**Organism**	**Hit gene name/ORF number**
	Ga0395992_01_182949_184436	22	Secreted	NP_953783.1	1.9E-7	34	*Geobacter*	*omcC omcBC orf2*
				YP_003639882	5.0E-30	28	*sulfurreducens*	
							*Thermincola potens*	TherJR_1117
Putative Q-oxidase	Ga0395992_01_187284_188528	5	Secreted	–				
Cytochromes encoded in the “pilin-cytochrome” cluster	Ga0395992_01_217646_219709	11	Secreted	NP_717047.1	1.8E-6	27	*Shewanella oneidensis*	*dmsE*
				YP_003639887	2.0E-12	28	*Thermincola potens*	TherJR_1122
	Ga0395992_01_220048_222057	11	Secreted	NP_951727.2	2.7E-6	33	*Geobacter sulfurreducens*	*omcX*
				YP_003639887	1.0E-17	25	*Thermincola potens*	TherJR_1122
	Ga0395992_01_222075_224618	9	Secreted	–				
	Ga0395992_02_29558_30178	6	Transmembrane, outer facing	YP_003639882	3.0E-22	30	*Thermincola potens*	TherJR_1117
	Ga0395992_02_30996_31874	7	Transmembrane, outer facing	NP_717386.1	4.0E-12	30	*Shewanella oneidensis*	*mtrA*
**OmhA**	Ga0395992_02_112574_114619	11	Secreted	ADG83432.1	4.0E-44	30	*Thermincola potens*	*ocwA*
**NrfA**	Ga0395992_02_129131_130396	5	Transmembrane, outer facing	–				
**NrfH**	Ga0395992_02_130419_130832	4	Secreted	ADG83432.1	2.0E-19	29	*Thermincola potens*	*ocwA*
Putative Q-oxidase	Ga0395992_02_135863_137242	20	Secreted	NP_953783.1	2.7E-6	29	*Geobacter sulfurreducens*	*omcC omcBC orf1 and 2*
				NP_953777.1	2.7E-6	29	*Thermincola potens*	TherJR_1117
				YP_003639882	2.0E-29	28		
**SmhA**	Ga0395992_02_137389_139104	8	Secreted	–				
	Ga0395992_02_147039_148391	14	Transmembrane, outer facing	YP_003639882	6.0E-57	33	*Thermincola potens*	TherJR_1117
	Ga0395992_02_151260_153788	12	Secreted	NP_719884.1	2.5E-6	45	*Shewanella oneidensis*	*mtrA*
	Ga0395992_01_477471_477917	4	Secreted	–				
**SmhC**	Ga0395992_02_433869_435545	7	Secreted	–				
**SmhB**	Ga0395992_03_307983_310529	12	Secreted	YP_003639887	7.0E-17	24	*Thermincola potens*	TherJR_1122
	Ga0395992_01_684739_686151	8	Secreted	–				
	Ga0395992_01_647313_648317	10	Transmembrane	YP_003639882	3.0E-81	42	*Thermincola potens*	TherJR_1117

*^1^Refer to the text for details. ^2^Tested with Phobius, SignalP, TMHMM, TatP 1.0, SecretomeP, PsortB.*

### Identification of Predominant Multiheme Cytochromes Produced by *C. ferrireducens* During the Growth With Insoluble Electron Acceptors

**FIGURE 4:**
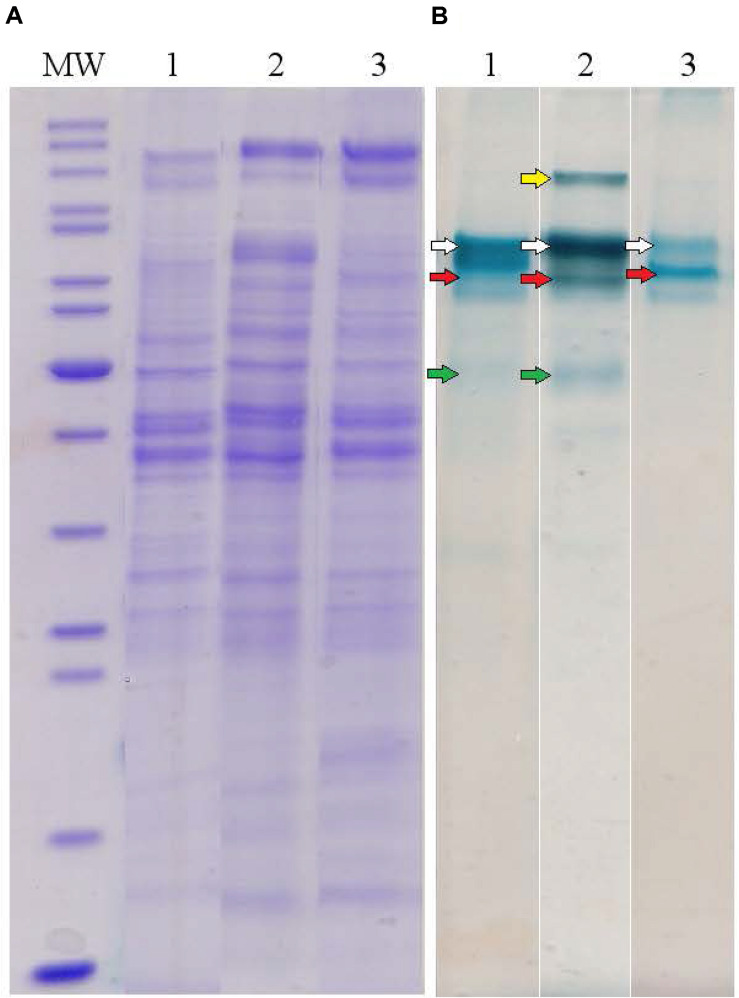
Protein profiles of crude cell extracts of *C. ferrireducens* grown in the presence of ferrihydrite and alternative soluble electron acceptors. **(A)** Lanes1–3 show full-length Coomassie-stained gels for proteins. **(B)** Lanes 1–3 show the same gels, benzidine-stained to visualize *c*-type hemes. Lane numbers indicate different electron acceptors: 1—ferrihydrite; 2—ferric citrate; 3—fumarate. MW—molecular weight markers (top to bottom: 200, 150, 120, 100, 85, 70, 60, 50, 40, 30, 25, 20, 15, 10 kDa); *white arrows* indicate heme bands containing OmhA cytochrome, *red arrows* SmhA, *yellow arrow* SmhB, and *green arrows* SmhC (refer to the text for cytochromes’ description). The Coomassie and benzidine stained gels received the same protein loading (13–14 μg per lane).

To determine major EET-related multiheme cytochromes of *C. ferrireducens*, the organism was cultivated at three different growth conditions: with ferrihydrite, soluble ferric citrate, or fumarate as electron acceptors. Cell-free extracts of the cells grown under these conditions were analyzed by SDS-PAGE with gels stained for protein and *c*-type hemes. In each heme-stained profile of crude cell extracts, two or three major and several minor heme bands were recovered (Figure 4). Molecular masses of heme-stained proteins ranged from 40 to 120 kDa. In-gel tryptic digests followed by LC-MS analysis of the major heme bands revealed, that the most intense bands of ca. 80 kDa, recovered at all the tested culture conditions, contain the cytochromes Ga0395992_02_112574_114619 and Ga0395992_02_137389_139104 with 11 and eight predicted heme-binding motifs, respectively. In the cells grown with ferrihydrite or ferric citrate, the third intensively heme-stained band of ca. 50 kDa was revealed, containing the heptaheme cytochrome Ga0395992_02_433869_435545. In the cells grown with soluble ferric citrate, the fourth heme band of the cytochrome Ga0395992_03_307983_310529 of 110 kDa with 12 predicted heme-binding motifs was revealed. No other multiheme cytochromes were identified in the major heme bands on the gels regardless of the growth condition used. Four multihemes detected in the most intense heme bands were designated according to their predicted subcellular localization (Table 1 and Supplementary Table S5): Ga0395992_02_112574_114619 was designated as OmhA (“Outer MultiHeme”), Ga0395992_02_137389_139104 as SmhA (“Secreted MultiHeme”), Ga0395992_03_307983_310529 as SmhB, and Ga0395992_02_433869_435545 as SmhC.

### Proteomic Profiling of *C. ferrireducens* Cells Grown With Different Electron Acceptors

**FIGURE 5:**
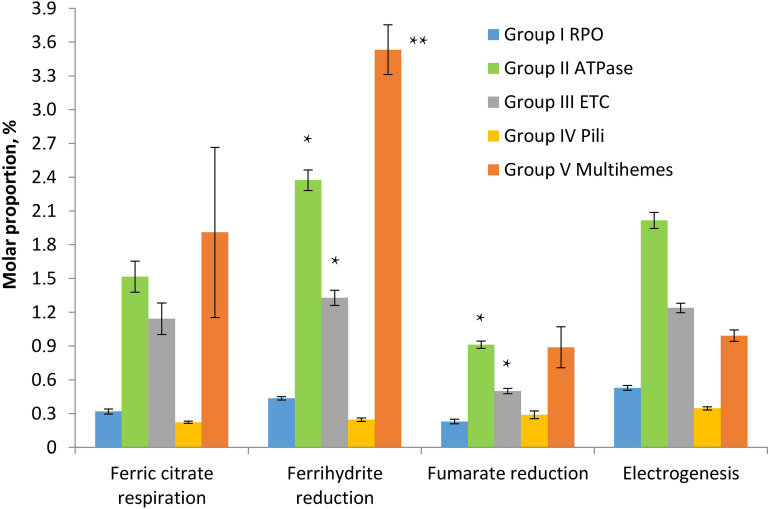
Molar proportions (riBAQs) of selected protein groups determining housekeeping, respiratory, and EET processes in *C. ferrireducens* cells grown with different electron acceptors. For each protein group, summarized molar proportion (riBAQ) of all its proteins is presented. Whole set of proteins detected in a sample is taken as 100%. Error bars indicate standard deviations in summarized riBAQs of each group. Asterisks indicate the most significant, statistically supported differences in molar proportions of protein groups (refer to the text for detail). *Electrogenic* growth conditions imply utilization of a stainless steel anode as the electron acceptor. The cells at all the growth conditions were harvested at the late logarithmic growth phase. Target proteins are grouped as follows: *Group I RPO*—four proteins of α, β, β’, and ω subunits of DNA-directed RNA polymerase, *Group II ATPase*—six proteins of α, β, γ, δ, ε, and B subunits of F_0_F_1_-type ATP synthase, *Group III ETC*—totally 17 proteins of A to F and H to N subunits of proton-translocating type I NADH-dehydrogenase together with four subunits of membrane-bound succinate dehydrogenase/fumarate reductase, *Group IV Pili*—10 proteins of pili assembly encoded in the “pilin-cytochrome” cluster (“PilA-C,” PilBT, PilVMNO proteins, and three other proteins with type-IV pilin N-terminal methylation sites), *Group V Multihemes*—10 multiheme cytochromes which are predicted to be secreted or cell surface-associated proteins (refer to the legend of Figure 6 for detail). The full set of the proteins included in each group appears in Supplementary Table S6.

**FIGURE 6:**
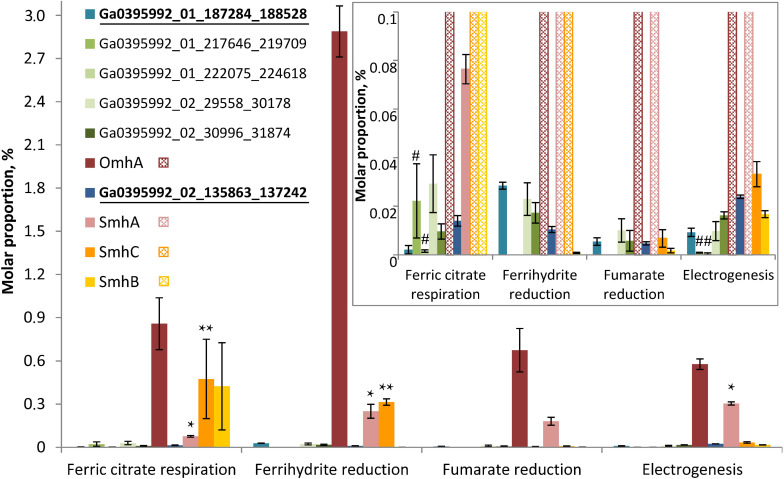
Molar proportions (riBAQs) of individual multiheme cytochrome proteins. The data are shown for all the putative EET-related cytochromes of *C. ferrireducens*, which were identified in protein profiles: OmhA, SmhA, SmhB, SmhC cytochromes, Ga0395992_01_217646_219709 and Ga0395992_01_222075_224618 encoded in the “pilin-cytochrome” cluster, as well as cytochromes Ga0395992_02_29558_30178, Ga0395992_02_30996_31874, and putative quinol-oxidizing cytochromes Ga0395992_01_187284_188528 and Ga0395992_01_684739_686151. *Inset*—molar proportions of minor cytochromes (with riBAQs below 0.1), predominating cytochromes are cross-hatched in the inset. Putative Q-oxidases (Ga0395992_02_135863_137242 and Ga0395992_01_187284_188528) are marked bold and underlined. Asterisks indicate significant, statistically supported differences, which are not visually obvious on the figure. Singular asterisks indicate the differences in molar proportions of SmhA cytochrome in the cells grown with ferric citrate, ferrihydrite, and on anode; double asterisks indicate the differences in SmhC representation in ferric citrate-reducing or ferrihydrite-reducing cells (refer to the text for detail). Hash symbols indicate the cytochromes Ga0395992_01_217646_219709 and Ga0395992_01_222075_224618, encoded in the “pilin-cytochrome” cluster and expressed only in electrogenic and ferric citrate-respiring cells. Clear differences in molar proportions (such as for OmhA in ferrihydrite-reducing and other cells) are not specially marked.

To assess the relevance of identified multihemes for EET and detect any accessory proteins involved in electron transfer to insoluble electron acceptors, the results of a shotgun proteomic analysis of crude extracts of cells, harvested at late logarithmic growth phase, were compared across four different cultivation conditions, each in three biological replicates: ferrihydrite reduction, electrogenesis, soluble ferric citrate reduction, and fumarate reduction. In each case, the triplicate samples were taken from three different cultures or BES systems, respectively. Tandem MS revealed 1524 different proteins identified with a minimum of two unique peptides. The number of proteins identified per each sample is provided in Supplementary Table S6. We summarized mole proportions (riBAQs) of selected marker proteins, which determine housekeeping, respiratory, and EET metabolic processes. Selected proteins were divided into five groups by their predicted functions: group I contains subunits of DNA-directed RNA polymerase, group II—subunits of F_0_F_1_-type ATP synthase, group III—complexes I and II of the respiratory ETC, group IV involves structural and accessory proteins of type-IV pili assembly encoded in the “pilin-cytochrome” cluster, group V contains all the multiheme cytochromes which were predicted to be secreted or cell surface-associated proteins and were identified in crude cell extracts or proteomic profiles. The full set of marker proteins from all the five groups is highlighted in Figures 5, 6 and in Supplementary Table S6. Expression profiling (Figure 5) revealed similar proportion of RNA polymerase proteins in all the tested cell preparations [individual (ind.) *P*-values in ANOVA pairwise comparisons ranged from 0.43 to 0.81, Supplementary Table S3]. Profiles of the ATP-ase and respiratory complexes of the ETC are likely to reflect fluctuations in the activity of oxidative phosphorylation pathways when respiring different electron acceptors. In particular, the highest and lowest molar proportions of ATP-ase and ETC complexes are observed in the cells grown with the most and the least energetically favorable electron acceptors of *C. ferrireducens*, ferrihydrite, and fumarate, respectively (Thauer et al., 1977; Gavrilov et al., 2012). Expression levels of ATP-ase and ETC complexes are, respectively, 45 and 69% higher in ferrihydrite-grown cells comparing to fumarate-grown ones. However, only ATP-ase overexpression is statistically supported (ind. *P*-value 0.0017, Supplementary Table S3) due to high expression dissimilarity of individual proteins from the ETC complex (Supplementary Table S6). The highest proportion of multiheme cytochromes is clearly observed in ferrihydrite-grown cells (Figure 5), 93–304% difference in molar proportions of this group proteins was quantified when comparing ferrihydrite respiration conditions with the others tested (ind. *P*-values < 0.0004, Supplementary Table S3).

Profiling of individual multiheme proteins uncovered the same four predominant cytochromes that were identified in SDS-PAGE gels (OmhA, SmhA, SmhB, SmhC, Figure 4). These cytochromes are constitutively expressed in *C. ferrireducens* cells but their expression level differs significantly depending on the electron acceptor supplied (Figure 6), that is supported by ordinary two-way ANOVA tests retrieving *F*(3,80) factor of 69.17 at a *P*-value < 0.0001 for electron acceptors as a source of variation (Supplementary Table S2). OmhA multiheme revealed the highest molar proportion among cytochromes at all the conditions tested with maximal share in ferrihydrite-grown cells (Figure 6), which is 237–400% higher than in other cells (ind. *P*-values < 0.15 for all the pairwise comparisons, Supplementary Table S4). SmhA cytochrome appeared to be less influenced by the electron acceptor utilized. Statistically supported upregulation was only observed in ferrihydrite-reducing and electrogenic cells comparing to ferric citrate-reducing ones (227 and 298% difference at ind. *P*-values 0.0099 and 0.0009, respectively). The cytochrome SmhC is clearly upregulated in the cells grown with Fe(III) compounds, both soluble and insoluble (from 846% difference in ferrihydrite-reducing vs. electrogenic cells to 6955% difference in citrate-reducing vs. fumarate-reducing cells at ind. *P*-values < 0.0001 for all pairwise comparisons, Supplementary Table S4). SmhB cytochrome is rather specific for the cells reducing soluble ferric citrate (Figure 6), its relative abundance increased by 2435% when comparing these cells with electrogenic ones, and by 25,000 and 57,000% when comparing with fumarate- and ferrihydrite-reducing cells, respectively, at ind. *P*-values < 0.0001 in all the cases (Supplementary Table S4). Among minor multihemes with molar proportion below 0.1 (Figure 6, *inset*), of note is constitutive production of putative quinol-oxidizing cytochromes Ga0395992_02_135863_137242 and Ga0395992_01_187284_188528, but not the CymA homolog Ga0395992_01_647313_648317. Also, a pair of adjacently encoded cytochromes Ga0395992_02_29558_30178 and Ga0395992_02_30996_31874, homologous to secreted periplasmic cytochromes of *Shewanella* (Supplementary Table S5), was expressed constitutively at a minor level. Surprisingly, the cytochromes Ga0395992_01_217646_219709 and Ga0395992_01_222075_224618, encoded in the “pilin-cytochrome” cluster, were only detected in electrogenic and ferric citrate-respiring cells and their expression profile across different electron acceptors (Figure 6, *inset*) did not correlate with that of the pilin assembly proteins (Figure 5).

### OmhA Cytochrome Distribution in the Fractions of Ferrihydrite-Reducing Cultures

Magnetite produced by *C. ferrireducens* culture was separated from the cells and further washed with SDS. The wash contained only eight proteins identified in tryptic digests by LC-MS/MS (Supplementary Table S7). Among those proteins the only cytochrome was OmhA. It was also the only cytochrome identified in the fraction of proteins, sheared from the cell surface by non-destructive shaking (Supplementary Table S7). This result correlates with predicted localization of OmhA on the cell surface and with our previous finding that *C. ferrireducens* loses ferrihydrite-reducing activity after shearing off the cell surface proteins (Gavrilov et al., 2012). SDS-PAGE-MS/MS analysis revealed that OmhA is recovered in major heme bands in both soluble and insoluble protein fractions of crude extracts of the cells grown with ferrihydrite (Supplementary Figure S5). OmhA detection in the soluble protein fraction and easy shearing of the cytochrome from the cells probably result from its weak binding to the cell surface. From the other hand, the abundance of OmhA cytochrome in the fraction of insoluble proteins (presumably, membrane-associated) and on the surface of cells and biogenic magnetite they produced supports our proposal for the key role of this multiheme protein in EET chain of *C. ferrireducens*.

### Phylogeny of the Major EET-Related Multiheme Cytochromes

**FIGURE 7:**
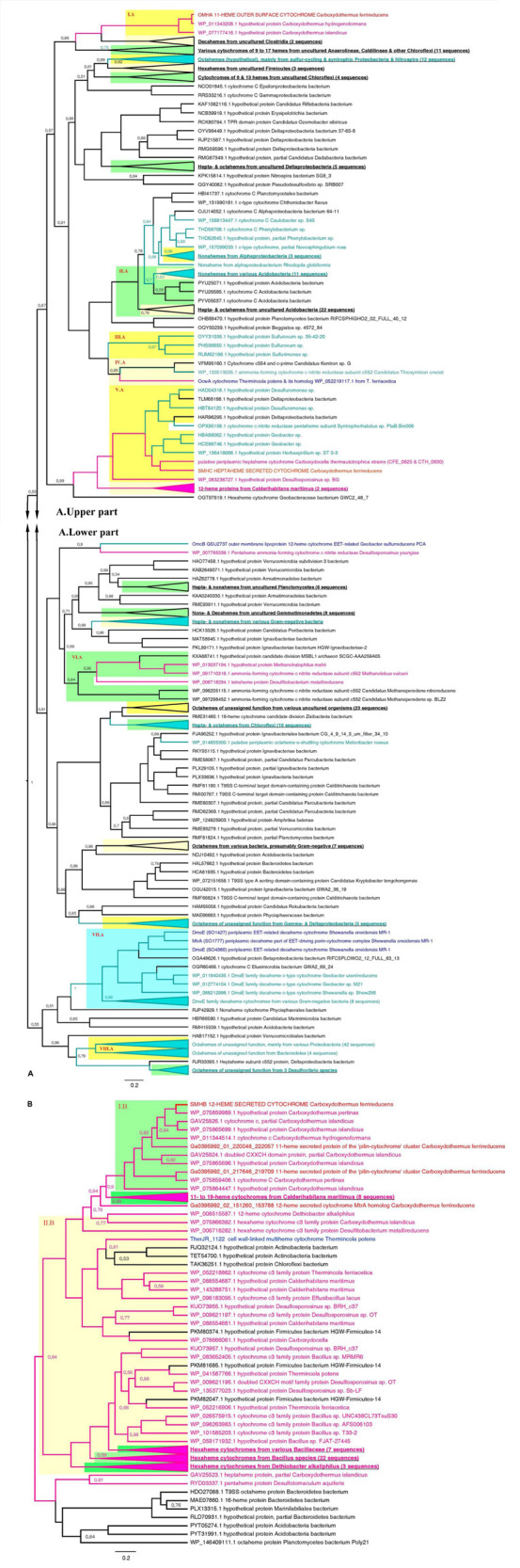
Consensus trees constructed after Bayesian inference of phylogeny from the MAFFT alignment of OmhA and SmhC **(A)**, and SmhB **(B)** cytochromes of *C. ferrireducens* and their best blast hits (see section “Materials and Methods” for detail). Unrooted 50% majority rule consensus phylograms are displayed as rectangular trees, for which posterior probability values are shown; posterior values of 1 are omitted for clarity. Mean branch lengths are characterized by scale bars indicating the evolutionary distance between the proteins (changes per amino acid position). The branches are annotated with labels indicating the protein sequence accession number, the protein name as retrieved from the database and the source organism. Branches and labels of the proteins are colored *pink* for the proteins retrieved from monoderm cultured organisms, *cyan* for the proteins from diderm cultured organisms, and *black* for the proteins from uncultured organisms. The target sequences of OmhA, SmhC, SmhB, and homologous cytochromes of *C. ferrireducens* are labeled *red* and their homologs from previously described EET-related cytochromes of other organisms are labeled *dark blue*. Clusters of sequences are categorized and highlighted where appropriate as follows. *Blue cluster* combines sequences of DmsE family decaheme cytochromes. *Light and deep yellow clusters* combine related sequences by the number of predicted heme-binding sites in them. *Green clusters* contain related sequences of various heme numbers from distinct taxonomic or physiological groups of source organisms. Highlighted clusters are labeled whether by collapsed subtrees with annotations given in bold underlined font or by red Roman numerals denoting the following. Cluster I.A—OmhA and related 11-heme proteins from *Carboxydothermus* spp.; cluster II.A—various multihemes from Acidobacteria; cluster III.A—octahemes from *Epsilonproteobacteria*; cluster IV.A—OcwA from “*T. potens*” and related nonaheme proteins; cluster V.A—SmhC and related hexa- and heptaheme proteins; cluster VI.A—9- to 16-heme proteins from monoderm prokaryotes, methanogenic archaea; cluster VII.A—see the *blue cluster* above; cluster VIII.A—octahemes of unassigned function from various Proteobacteria and Bacteroidetes; cluster I.B—SmhB and its homologs from *Carboxydothermus* species; cluster II.B—hexaheme proteins with unassigned function from various Firmicutes.

Phylogenetic analysis of OmhA, SmhA, SmhB, and SmhC protein sequences revealed their relationship with diverse groups of cytochromes retrieved from both monoderm and diderm prokaryotes. Since the majority of these multihemes have no functional assignment yet, this blurs evolutionary traits of *C. ferrireducens*’ proteins. Their distant relatedness with previously described EET-driving multihemes is clear (Figure 7 and Supplementary Figure S6) as well as the more recent evolutionary history of the SmhA and SmhB cytochromes. The octaheme SmhA falls into a clade sharing the root with a large clade of octaheme tetrathionate reductases (Otr) from Gram-negative bacteria (Supplementary Figure S6). Dodecaheme SmhB and the 11-heme cytochrome Ga0395992_01_217646_219709, both of which are strongly upregulated under ferric citrate respiration (10^3^–10^4^% differences when comparing with other culture conditions, ind. *P*-values < 0.0001, Supplementary Table S4 and Figure 6), belong to a clade, containing two other multihemes of *C. ferrireducens* and branching from a group of bacillary hexahemes (Figure 7B). More obscure is the phylogeny of OmhA and SmhC cytochromes, both upregulated upon ferrihydrite respiration (Figure 6). Those appear to be related to each other and belong to different subclades of proteins from Gram-positive bacteria containing 11 or 6–7 hemes, respectively (Figure 7A). In contrast, their distant relative, OcwA cytochrome of “*T. potens*,” is the only protein from a monoderm organism in a subclade of nonahemes retrieved from Gram-negative bacteria (Figure 7A). However, three distinct clades enclosing the cytochromes OmhA, SmhC, and OcwA, respectively, share common roots with diverse cytochromes possessing 5–17 heme-binding sites and retrieved from various organisms, mainly uncultured.

## Discussion

Direct electron exchange between microorganisms and redox-active minerals plays a pivotal role in the biogeochemical cycles of metals. Understanding mechanisms of electron flow, especially those evolved in thermophilic Fe(III)-reducing prokaryotes closely related to ancestral forms of life, is essential for gaining insights into the processes that affected Earth’s surface mineralogy in the Eoarchean Era and continue to influence it at present (Hazen et al., 2008). Our observations of magnetite crystallizing activity and magnetite-facilitated exoelectrogenesis in the cultures of the thermophile *C. ferrireducens* support this notion. Active colonization of ferrihydrite by *C. ferrireducens* (Figure 1 and Supplementary Figure S1), ferrihydrite transformation into unusually structured large magnetite crystals (Figure 2 and Supplementary Figure S3) by growing cultures, and previously reported correlation between kinetics of microbial growth and Fe(III) reduction (Gavrilov et al., 2012) suggest biocontrolled formation of the novel mineral phase. This control could be realized via electron exchange between the mineral and redox enzymes, localized on the cell surface.

We previously reported outer surface *c*-type cytochromes to be important for ferrihydrite reduction in *C. ferrireducens* (Gavrilov et al., 2012). In the current work, we have identified candidate genes encoding such cytochromes and revealed the multiheme proteins with strongly upregulated expression in ferrihydrite-reducing cells. The most abundant of these cytochromes (OmhA) is predicted to be localized on the cell surface in a strategic position to provide reductive foci for direct interaction with the outermost Fe(III) atoms of ferrihydrite.

Detection of the OmhA protein in the cell-to-mineral interface, i.e., among the proteins sheared from surfaces of cells or biogenic magnetite (Supplementary Table S7 and Supplementary Figure S5), indicate weak binding of the cytochrome to the cell envelope. We propose that this allows it to diffuse from the cell and rendezvous with remote mineral nanoparticles, donating electrons to them (as predicted for external cytochromes MtrC and OmcA of *S. oneidensis*) (Subramanian et al., 2018). Accordingly, the OmhA cytochrome could function either as a direct electron conduit, connecting the cellular energy transducing system with the extracellular mineral electron acceptor, or as an electron shuttle inside restricted volumes of the mineral cavities near the cell surface (shown in Figure 2A), where the losses of secreted compounds are limited, and the protein could transfer electrons to remote mineral particles inaccessible for cell surface structures.

Additionally, the EET pathway in *C. ferrireducens* could indirectly involve pili-like filamentous appendages forming complex networks, which connect the cells with Fe-containing nanoparticles (Figure 1C). These proteinaceous structures are non-conductive (Supplementary Figure S2) and rather serve for cell-to-mineral anchoring or for aligning the mineral nanoparticles together along the filaments in microenvironments surrounding the cells. This function could be important during initial steps of colonial growth and nucleation of magnetite crystals within the colonies. During further “big scale” crystallization, the redox mineral becomes more readily accessible to the cell surface cytochromes.

Micron-scale magnetite crystals can serve as more effective mediators of direct interspecies electron transfer than nanoparticles in natural anoxic sedimentary environments. The electrons could be tunneled from a single reductive focus on the cell surface of *C. ferrireducens* to the bulk of the conductive mineral and further, to several symbiotic organisms, which could utilize the same magnetite crystal as the electron donor, as methanogens (Shi et al., 2016; Zhuang et al., 2018) or anoxigenic phototrophs (Byrne et al., 2015) do. Thus, enhancing the formation of large mineral structures is likely to be energetically advantageous for Fe(III)-respiring microbial colonies. Exoelectrogenic activity of *C. ferrireducens* (Figure 3) would allow the organism to discharge electrons to the conductive biogenic mineral. The proposed efficiency of electron transfer out of *C. ferrireducens* cells through magnetite crystals is supported by a stabilizing (Figure 3A) effect of ferrihydrite particles on electrogenic activity of the organism. Previously, several reports indicated such an effect of solid-phase Fe(III/II) nanoparticles in mesophilic MFCs (Kato et al., 2010, 2012).

Interestingly, among the wide diversity of cultured prokaryotes capable of EET, only Gram-positive Fe(III) reducing clostridia of the genus *Thermoanaerobacter* have been reported to induce formation of micron-scale magnetite crystals (Zhang et al., 1998; Li, 2012). In contrast, nanoparticulate biogenic magnetite forms are well characterized in Fe(III) reducers of various phylogeny and physiology, and the microorganisms are regarded to have little, if any, control over nanoparticles biomineralization (Jimenez-Lopez et al., 2010). The first report on large biogenic magnetite structures described 15–40 μm octahedron and cubic crystals recovered from a thermophilic enrichment dominated by Firmicutes (Slobodkin et al., 1995). Internal structure of this mineral has not been studied, and no imprints or invasion of bacterial cells have been observed so far in this (Slobodkin et al., 1995) or any other (Zhang et al., 1998; Jimenez-Lopez et al., 2010; Li, 2012) biogenic magnetites. Our results provide the first observations of these fascinating minerals. Considering the abundance of Gram-positive bacteria among thermophiles capable of EET (Wrighton et al., 2008), their ability to enhance magnetite crystallization and benefit from this process may increase the overall efficiency of energy conservation from metal reduction in thermophilic microbial communities compared to their mesophilic counterparts.

Predictably, the most probable determinants of EET we have identified in *C. ferrireducens* by genomic and proteomic approaches (Figure 6 and Supplementary Table S5) appear to be multiheme *c*-type cytochromes. This finding supports previous proposals on global distribution of multiheme-related EET pathways throughout the prokaryotic world (Shi et al., 2016) and argues against an alternative concept on flavin-based cytochrome-independent EET in Gram-positive mesophiles (Light et al., 2018; Pankratova et al., 2019). Indeed, in our experiments at all the tested culture conditions, the total proportion of secreted or cell-surface associated multiheme cytochromes was comparable with that of ATP-ase or ETC complexes, pointing out the equivalent significance of the multihemes and basic components of oxidative phosphorylation pathways for the metabolism of *C. ferrireducens* during its growth with Fe(III) compounds, anodes, and even soluble electron acceptors (Figure 5). Low expression of multihemes in electrogenic cells comparing to those grown with ferrihydrite (-78% difference, ind. *P*-value < 0.0001) probably reflects their biofilm lifestyle (Figures 3B,C and Supplementary Figures S4A–C), which imposes spatial constraints on the distribution of terminal oxidoreductases over the cell surface. The cells in the biofilm, immobilized on the anode surface, contact this electron acceptor with a restricted area of their envelope. Only the cytochrome molecules located in this restricted area remain useful for anode respiration. In our experiments, the highest current density generated by anode-respiring *C. ferrireducens* cells was comparatively low (68 mA/m^2^), indicative of one layer (≤2 μm) thick electrogenic biofilm, suggesting EET via direct contact of cell wall-associated cytochromes with the anode (Torres et al., 2010). In contrast, ferrihydrite-reducing cells, which are surrounded by nanoparticles or larger crystal structures of their electron acceptor (Figures 1, 2), could employ larger quantities of multiheme molecules, distributed all over the cell surface, to get advantage of using all the available cell-to-mineral contact points for electron transfer to Fe(III). Surprisingly, the cytochromes encoded in the proposed “pilin-cytochrome” cluster had negligible expression levels in all the tested cultures. This correlates with the absence of conductivity in pili-like filaments inferred both from PilA sequence analysis and CP-AFM measurements (Supplementary Figure S2). However, the observed production of filamentous networks in magnetite-forming *C. ferrireducens* cultures (Figures 1C,D) necessitates further investigation of the nature and physiological role of these extracellular filaments.

Multiheme cytochromes profiles, and particularly, constitutive production and the strongest positive expression response of OmhA to ferrihydrite (237–400% difference comparing to other culture conditions, ind. *P*-values < 0.0001, Supplementary Table S4 and Figure 6), allow us to conclude that this cytochrome is the key protein for EET to ferrihydrite and is likely to be the major multiheme protein, determining the reduction of other electron acceptors tested, including stainless steel anodes. At the same time, differential expression of putative secreted SmhB and SmhC cytochromes in response to the electron acceptor utilized indicates modularity of the respiratory pathway in *C. ferrireducens*, which implies constitutive activity of core electron transferring multihemes, such as OmhA, SmhA, and putative quinol-oxidizing cytochromes Ga0395992_01_187284_188528 and Ga0395992_02_135863_137242, supplemented with the activity of specific multihemes targeting electrons to different electron acceptors. The prevalence of OmhA and SmhA multihemes among other cytochromes at three of the four culture conditions tested supports probable involvement of these proteins in several EET pathways. Indeed, as illustrated by Figure 6, OmhA had the highest molar proportion among multiheme cytochromes at all the culture conditions, ranging from 0.577% in electrogenic cells to 2.89% in ferrihydrite-respiring cells. SmhA had the second biggest molar proportion among cytochrome proteins in electrogenic and fumarate-respiring cells (0.304 and 0.180, respectively) and was almost equally represented with SmhC cytochrome in ferrihydrite-reducing cells (0.250 and 0.314% for SmhA and SmhC, respectively).

High expression level of OmhA and SmhA oxidoreductases could be sustained constitutively by a regulome of *C. ferrireducens* in order to compensate for electron acceptors that fluctuate rapidly in their availability in the natural environment of the organism. Such a metabolic strategy could represent a widespread response of microorganisms to sharp changes in the availability of electron acceptors. In particular, constitutive expression has been reported for different terminal oxidoreductases of a thermophilic iron-reducer *Melioribacter roseus* from a deep subsurface aquifer (Gavrilov et al., 2017), as well as for several multihemes involved in electron transfer to different electron acceptors in a mesophilic iron-reducing soil bacterium *Geobacter soli* (Cai et al., 2018). A modular principle of the respiratory system organization, which implies differential activity of various multihemes, has been previously proposed for *S. oneidensis* (Coursolle and Gralnick, 2010) and *Geobacter* species (Butler et al., 2010; Aklujkar et al., 2013; Cai et al., 2018). However, the key proteins of the EET chain in *C. ferrireducens* differ significantly from their previously described counterparts of *Geobacter*, *Shewanella*, and *Thermincola* species (Figure 7 and Supplementary Figure S6). Even putative terminal reductases OcwA from “*T. potens*” (Costa et al., 2019) and OmhA from *C. ferrireducens*, sharing considerable sequence similarity (Table 1 and Supplementary Table S5), fall into different clades of multihemes which diverged early from their common ancestor (Figure 7A). Moreover, it remains unclear if this ancestor protein belonged to a monoderm or diderm prokaryote.

Our phylogenetic reconstructions did not reveal any distinct evolutionary traits which have been recently proposed for EET-related cytochromes of thermophilic monoderm prokaryotes (Lusk, 2019). Instead, our data correlate with previous statements that gene truncation or duplication followed by sequence divergence and active horizontal gene transfer is the main mechanism of multiheme cytochromes evolution which is rather independent of the cell envelope structure of an organism and is mainly driven by its metabolic needs (Sharma et al., 2010; Kern et al., 2011). In the case of *C. ferrireducens*, an ancestor octaheme protein of SmhA cytochrome is likely to be acquired by horizontal gene transfer from a diderm bacterium (Supplementary Figure S6), while 12-heme SmhB cytochrome seems to originate from an ancestral monoderm bacterium by gene duplication and further vertical evolution (Figure 7B). Evolutionary history of OmhA and SmhC proteins is more complicated and probably involves multiple events driven by the need of *Carboxydothermus* species and their ancestors to effectively utilize high-potential insoluble electron acceptors, such as Fe(III) minerals.

## Conclusion

Newly identified secreted multiheme cytochromes OmhA and SmhA in *C. ferrireducens* have been shown to be involved in ferrihydrite to magnetite transformation and exoelectrogenesis, while a distinct cytochrome SmhB is likely involved in the reduction of soluble Fe(III). All these cytochromes are not associated with porin–cytochrome complexes or pilin-cytochrome assemblies, are phylogenetically distinct from the known external EET-driving cytochromes and thus represent novel extracellular electron transferring proteins. Further structural and functional characterization is planned to trace and reconstruct the pathways for cell-to-mineral electron flow in thermal environments inhabited by diverse thermophiles.

## Data Availability Statement

The datasets presented in this study can be found in online repositories. The names of the repository/repositories and accession number(s) can be found in the article/ Supplementary Material.

## Author Contributions

SG and EB-O convened the research. SG, DZ, and IE designed and performed cultivation experiments and mineral phase analysis. VP, TT, and ND designed and performed protein separation and identification experiments. JL and DK designed and performed the experiments on cytochromes identification in microbe-to-magnetite interphase. ME-N, KL, and SP designed and performed SEM, FIB, and conductive AFM studies. MZ analyzed proteomic data. OB designed and performed MFC experiments. SG and FR analyzed genomic data. SG and IE performed phylogeny reconstructions. SG, DZ, TT, JL, ME-N, FR, OB, and EB-O analyzed the data and wrote the manuscript. All authors contributed to the article and approved the submitted version.

## Conflict of Interest

The authors declare that the research was conducted in the absence of any commercial or financial relationships that could be construed as a potential conflict of interest.

## Footnotes

1 www.graphpad.com

2 http://weizhongli-lab.org/cdhit_suite/cgi-bin/index.cgi

3 https://img.jgi.doe.gov/

4 http://proteomecentral.proteomexchange.org
