# Supplementary material for: Novel Extracellular Electron Transfer Channels in a Gram-Positive Thermophilic Bacterium
Source: Front Microbiol. 2021 Jan 11;11:597818. doi: 10.3389/fmicb.2020.597818 (PMC7829351; doi:10.3389/fmicb.2020.597818)
Supplement: Supplementary file 4 [file Data_Sheet_1.pdf]

Novel extracellular electron transfer channels in a Gram-positive thermophilic Bacterium

Gavrilov S.N., Zavarzina D.G., Elizarov I.M., Tikhonova T.V., Dergousova N.I., Popov V.O., Lloyd J.R., Knight D., El-Naggar M.Y., Pirbadian S., Leung K.M., Robb F.T., Zakhartsev M.V., Bretschger O. and **Bonch-Osmolovskaya E.A.**

Supplementary materials

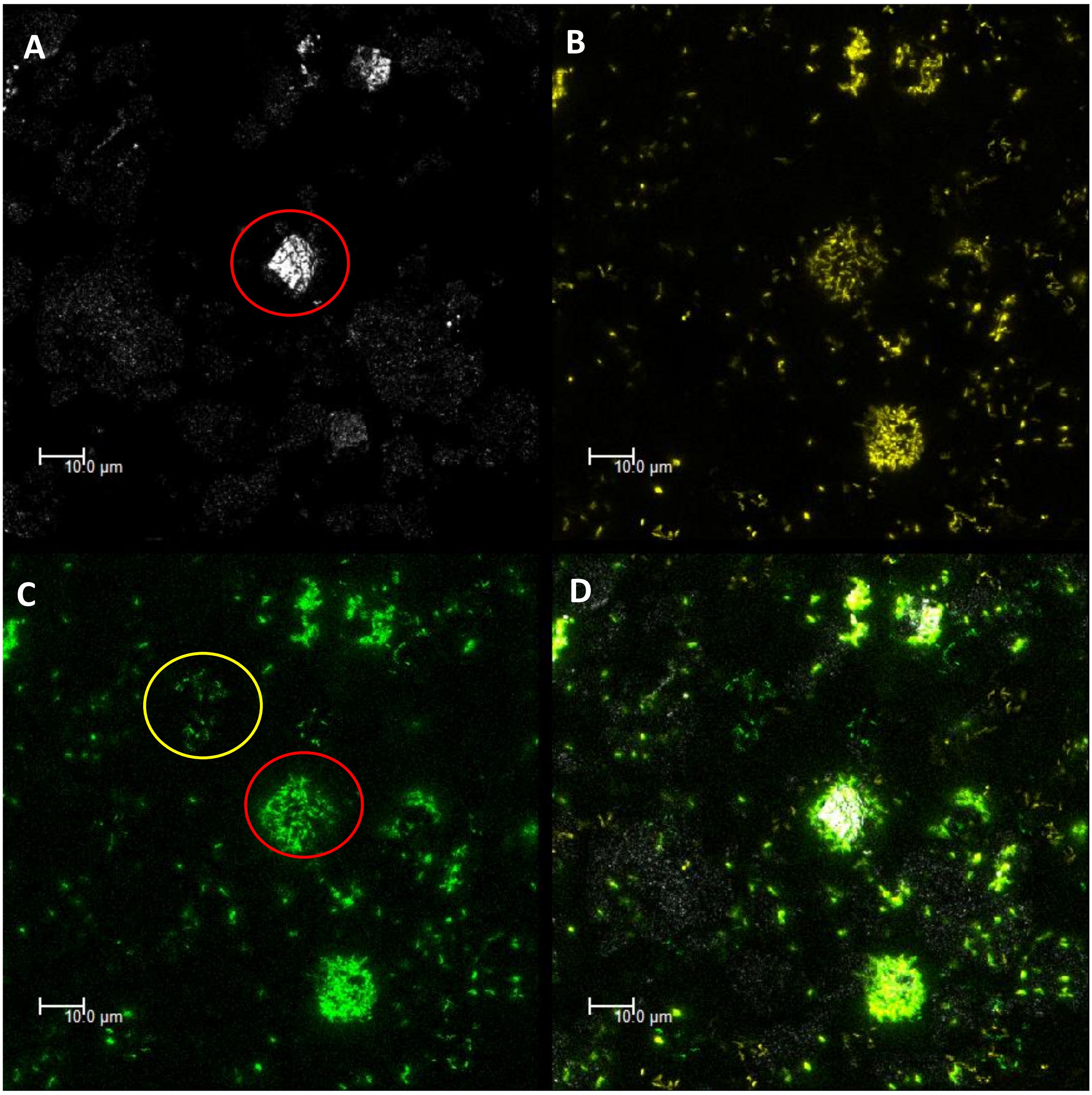

Supplementary figure S1.

Fig. S1. Confocal images of *C. ferrireducens* biofilms grown on ferrihydrite. Transmitted light dark-field (A) and fluorescent (B, C) images of a culture of *C. ferrireducens* grown with ferrihydrite. The culture samples were subsequently stained with Nano-Orange protein stain (B) and SYBR Green DNA stain (C). On the panel (D) superposition of (A) and (C) is presented. The same microscopic field, imaged from the same height at the same magnification is given at all the images. Bright objects at (A) and (D) correspond to magnetite particles formed by the culture. Note two types of cell aggregates (red and yellow circles at C) differing by their size and the number of cells. Larger cell aggregates (red circles) are colocated with large, micron-scale mineral particles.

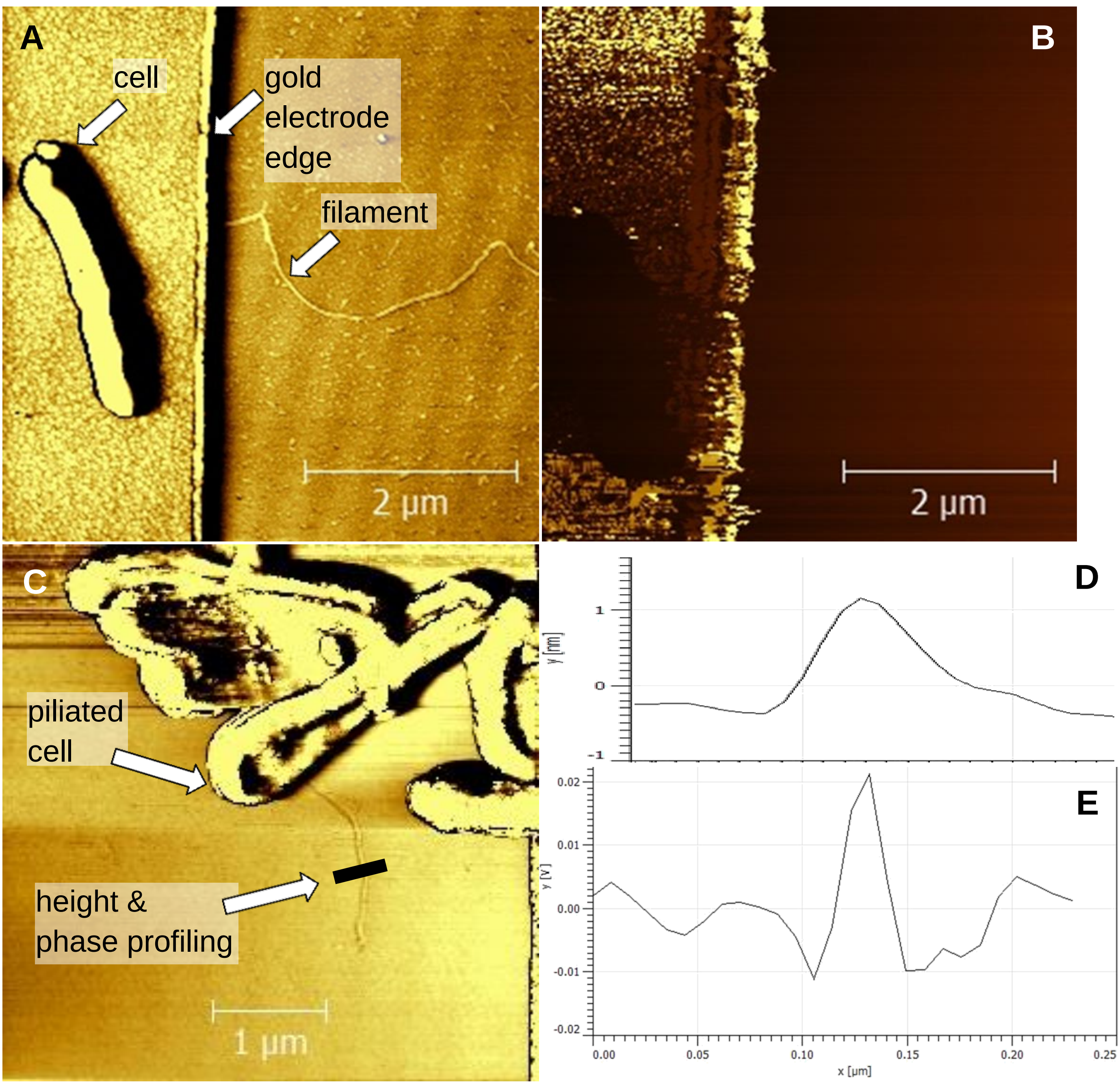

Supplementary figure S2

Fig. S2. Conducting probe atomic force (A and B) and scanning conductance (C-E) microscopy of pili-like filaments produced by *C. ferrireducens* cells grown with ferrihydrite. Topography (A) and current image (B) of a bacterial cell and its filament separated by the edge between a gold electrode (left) and SiO_2 (right). Note black (non-conductive) areas at (B) corresponding to the sites of the cell and the filament at (A). Scanning conductance microscopy (tapping phase in Lift mode) image (C) of a cell aggregate and a filament attached to the cell, height (D) and phase (E) profiles which were taken across the filament as marked by the black line on (C) and aligned along the same X-axis. Note positive phase shift at the phase profile (E) of the filament indicating the absence of conductivity.

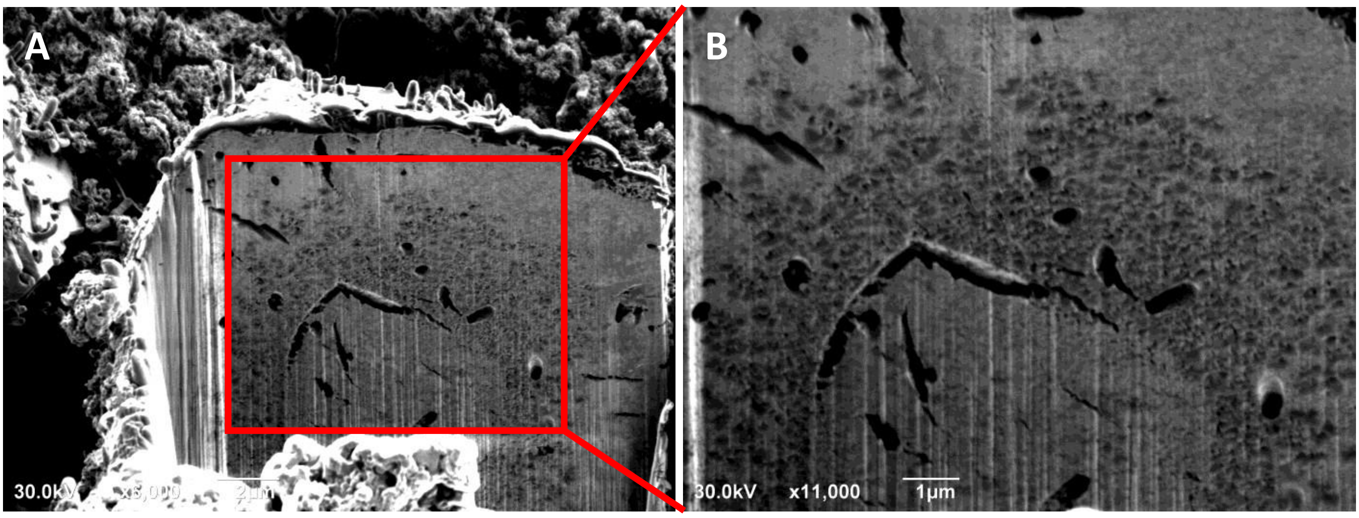

Supplementary figure S3

Fig. S3. (*A*) **The internal structure of a large biogenic magnetite crystal exposed by FIB-milling.** (*B*) Enlarged view of the red-bounded area of (*A*) revealing previous incorporation of cells within the crystal (note smooth contours of cell-like cavities in the bulk of the mineral).

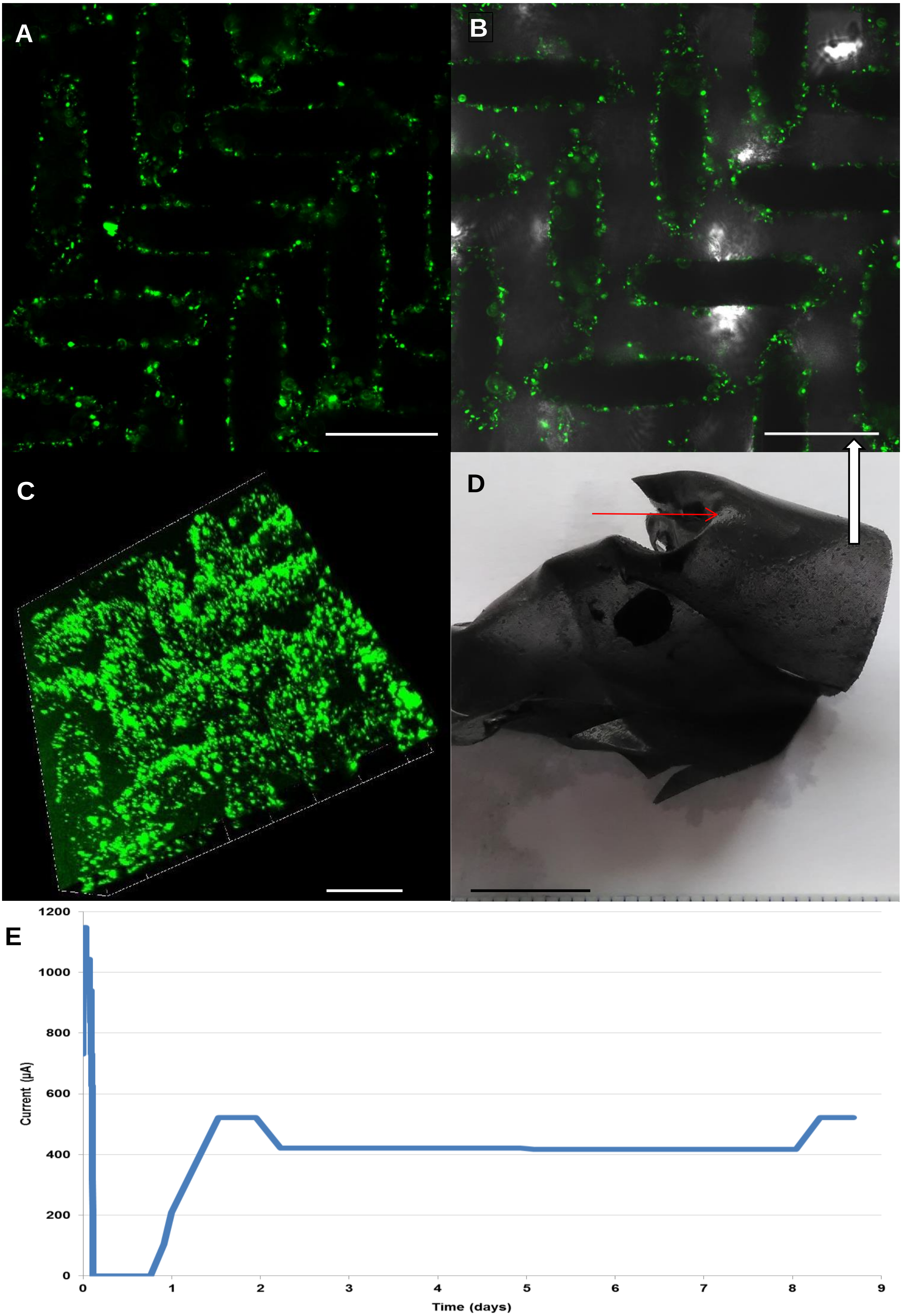
A
B
C
D
E
1200
1000
800
600
400
200
0
Current (µA)
0
1
2
3
4
5
6
7
8
9
Time (days)

Supplementary figure S4 (see above)

Fig. S4. (*A*) confocal fluorescent image of *C. ferrireducens* biofilm grown on a meshy stainless steel anode in a potentiostatic mode BES; *(B)* superposition of fluorescent and transmitted light images of the biofilm (note the meshy structure of the electrode traced by fluorescently stained cells); (*C*) 3D graphical sketch of the biofilm constructed from layerwise fluorescent images; *(D)* photo of a piece of stainless steel anode before confocal imaging; *(E)* current vs. time of a potentiostatic BES inoculated with *C. ferrireducens* and glycerol as the electron donor.

Same spot of the biofilm is represented on *(A)*, *(B)* and *(C)*. 3D sketch on *(C)* is constructed using Zen blue software from Carl Zeiss. *Red arrow* on *(D)* indicate a magnetite inclusion in the biofilm. *White arrow* on *(D)* indicate a spot of the biofilm imaged on *(A)*, *(B)* and *(C)*. Note the meshy texture of the biofilm. Scale bars on *(A)*, (*B*) and (*C*) are 50 µm, on (D) – 1 cm.

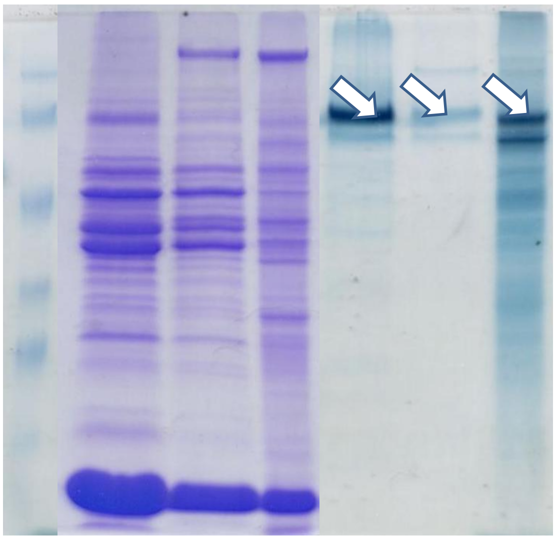

Supplementary figure S5

Fig. S5. Protein profiles of subcellular fractions of *C. ferrireducens* cells grown with ferrihydrite. Lanes 1 to 3 correspond to Coomassie-stained fractions for protein, lanes 4 to 6 correspond to the same protein preparations stained with benzidine to visualize *c*-type hemes. Two SDS-PAGE gels (Coomassie-stained and benzidine-stained) are superposed on the same image. The Coomassie- and benzidine-stained gels received the same protein loading. Lanes 1 and 4 – crude cell extract after cell disruption by ultrasonication, lanes 2 and 5 – soluble protein fraction after centrifugation of the crude cell extract at 100000 g, 40 min, lanes 3 and 6 – insoluble protein fraction obtained in the same procedure. MW – molecular weight markers (top to bottom: 117, 85, 48, 34, 26, 19 kDa); *white arrows* indicate heme bands containing OmhA cytochrome (as detected by MALDI-TOF MS analysis of protein-stained bands).

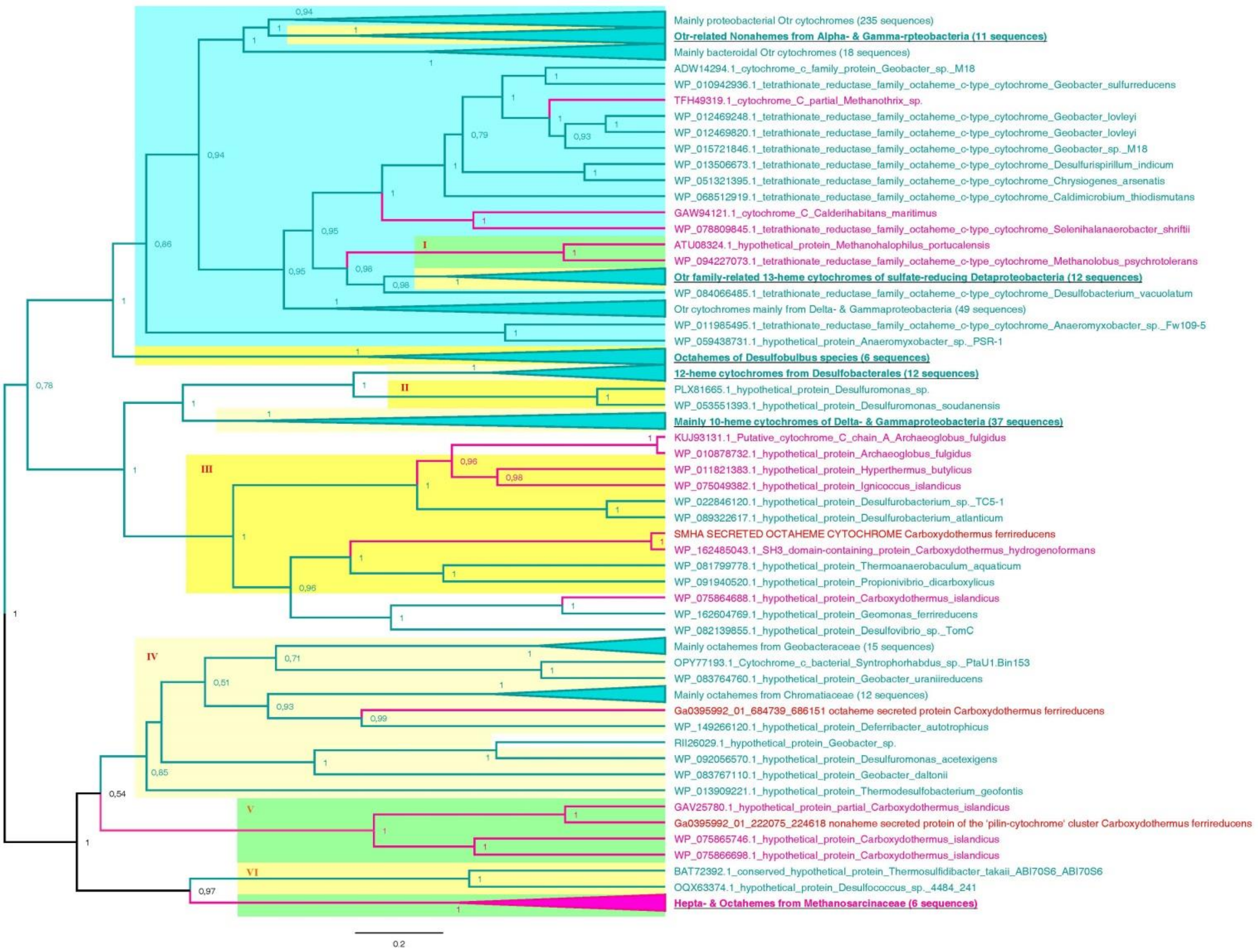

Supplementary figure S6.

Fig. S6. Consensus tree constructed after Bayesian inference of phylogeny from the MAFFT alignment of SmhA cytochrome of *C. ferrireducens* and its best blast hits (see *Methods* section for details). An unrooted 50% majority rule consensus phylogram was displayed as a rectangular tree, for which posterior probability values are shown. Mean branch lengths are characterized by a scale bar indicating the evolutionary distance between the proteins (changes per amino acid position). The branches are annotated with labels indicating the protein sequence accession number, the protein name as retrieved from the database and the source organism. Branches and labels of the proteins are colored according to the cell envelope structure of the source organisms: *pink* for the proteins from monoderm prokaryotes, *cyan* for the proteins from the diderms. The sequences of SmhA and related cytochromes from *C. ferrireducens* are labeled *red*. Clusters or subclusters of sequences are categorized and highlighted where appropriate as follows. *Blue cluster* combines sequences of octaheme tetrathionate reductase (Otr) family proteins including those containing additional heme-binding sites apart from the Otr octaheme domain, some subtrees of typical Otr sequences are collapsed for clarity. *Light and deep yellow clusters* combine related sequences by the number of predicted heme-binding sites in them, for the majority of the proteins in these clusters function is unassigned. *Green clusters* contain related sequences from distinct taxonomic groups of source organisms irrespectively of the number of hemes and function assignments of the proteins. Highlighted clusters are labeled whether by collapsed subtrees with annotations given in bold underlined font or by red Roman numerals denoting the following. Subcluster I – Otr-family cytochromes from archaea of *Methanosarcinaceae* family; cluster II – 11-heme cytochromes from *Desulfuromonas* species; cluster III – SmhA cytochrome and related octahemes with unassigned functions (hypothetical proteins); cluster IV - Ga0395992_01_684739_686151 cytochrome of *C. ferrireducens* and related octahemes with

unassigned functions; cluster V – related sequences of hepta- and octahemes from different *Carboxydothermus* species; cluster VI – deeply rooted heptahemes of Gram-negative bacteria.

Supplementary tables

Table S1. The results of ordinary two-way ANOVA test[1] for five protein groups[2] determining housekeeping, respiratory and EET processes in *C. ferrireducens*.

Source of Variation	% of total variation	P value		Significant?	
Interaction	4.9	0.0017		Yes	
protein group	12.75	<0.0001		Yes	
e-acceptor	2.779	0.0005		Yes	
ANOVA table	SS (Type III)	DF	MS	F factors (DFn, DFd)	P value
Interaction	1.729	12	0.1441	F (12, 526) = 2.676	P=0.0017
protein group	4.501	4	1.125	F (4, 526) = 20.90	P<0.0001
e-acceptor	0.9807	3	0.3269	F (3, 526) = 6.070	P=0.0005
Residual	28.33	526	0.05385		

[1] Refer to *Materials and Methods. Statistical Analysis of Proteomic Data* section for detailed description of the test parameters.
[2] Refer to the legend of Fig. 5 for detailed description of protein groups.

Table S2. The results of ordinary two-way ANOVA test[1] for individual multiheme cytochrome proteins[2] of *C. ferrireducens*.

Source of Variation	% of total variation	P value		Significant?	
Interaction	37.09	<0.0001	****	Yes	
cytochrome	56.28	<0.0001	****	Yes	
e-acceptor	4.780	<0.0001	****	Yes	
ANOVA table	SS (Type III)	DF	MS	F factors (DFn, DFd)	P value
Interaction	10.46	27	0.3875	F (27, 80) = 59.64	P<0.0001
cytochrome	15.88	9	1.764	F (9, 80) = 271.5	P<0.0001
e-acceptor	1.348	3	0.4495	F (3, 80) = 69.17	P<0.0001
Residual	0.5198	80	0.006498		

[1] Refer to *Materials and Methods. Statistical Analysis of Proteomic Data* section for detailed description of the test parameters.
[2] Refer to the legend of Fig. 6 for the description of multiheme cytochrome proteins considered for the analysis.

Table S3. Pairwise comparisons of representation of each of five protein groups, determining housekeeping, respiratory and EET processes in *C. ferrireducens*, at each of cultured conditions tested (four different electron acceptors).

Pairs (by protein group number[1] and electron acceptor utilized[2])	Mean Diff.	Discovery?	q value	Individual P Values
I:C vs. I:Fe	-0.02920	No	0.6436	0.7580
I:C vs. I:Fu	0.02229	No	0.6660	0.8141
I:C vs. I:E	-0.05240	No	0.5456	0.5804
I:C vs. II:C	-0.1731	No	0.0927	0.0458

I:C vs. II:Fe	-0.3159	Yes	0.0011	0.0003
I:C vs. II:Fu	-0.07228	No	0.4618	0.4037
I:C vs. II:E	-0.2565	Yes	0.0090	0.0032
I:C vs. III:C	0.009650	No	0.6934	0.8973
I:C vs. III:Fe	0.001410	No	0.7130	0.9849
I:C vs. III:Fu	0.04769	No	0.5126	0.5255
I:C vs. III:E	0.006648	No	0.6982	0.9289
I:C vs. IV:C	0.05729	No	0.4893	0.4701
I:C vs. IV:Fe	0.05503	No	0.4970	0.4878
I:C vs. IV:Fu	0.05072	No	0.5126	0.5226
I:C vs. IV:E	0.04498	No	0.5436	0.5706
I:C vs. V:C	-0.1114	No	0.2739	0.1604
I:C vs. V:Fe	-0.3620	Yes	<0.0001	<0.0001
I:C vs. V:Fu	-0.03148	No	0.6067	0.7014
I:C vs. V:E	-0.01962	No	0.6644	0.8046
I:Fe vs. I:Fu	0.05149	No	0.5456	0.5870
I:Fe vs. I:E	-0.02320	No	0.6644	0.8066
I:Fe vs. II:C	-0.1439	No	0.1727	0.0967
I:Fe vs. II:Fe	-0.2867	Yes	0.0034	0.0010
I:Fe vs. II:Fu	-0.04308	No	0.5635	0.6186
I:Fe vs. II:E	-0.2273	Yes	0.0213	0.0088
I:Fe vs. III:C	0.03885	No	0.5533	0.6034
I:Fe vs. III:Fe	0.03061	No	0.5968	0.6811
I:Fe vs. III:Fu	0.07689	No	0.3899	0.3061
I:Fe vs. III:E	0.03585	No	0.5667	0.6304
I:Fe vs. IV:C	0.08650	No	0.3717	0.2757
I:Fe vs. IV:Fe	0.08423	No	0.3778	0.2884
I:Fe vs. IV:Fu	0.07992	No	0.3948	0.3138
I:Fe vs. IV:E	0.07418	No	0.4257	0.3498
I:Fe vs. V:C	-0.08222	No	0.3858	0.3001
I:Fe vs. V:Fe	-0.3328	Yes	0.0003	<0.0001
I:Fe vs. V:Fu	-0.002280	No	0.7117	0.9778
I:Fe vs. V:E	0.009580	No	0.6935	0.9038
I:Fu vs. I:E	-0.07469	No	0.4703	0.4308
I:Fu vs. II:C	-0.1954	No	0.0538	0.0242
I:Fu vs. II:Fe	-0.3382	Yes	0.0004	0.0001
I:Fu vs. II:Fu	-0.09457	No	0.3717	0.2747
I:Fu vs. II:E	-0.2788	Yes	0.0043	0.0013
I:Fu vs. III:C	-0.01264	No	0.6775	0.8658
I:Fu vs. III:Fe	-0.02088	No	0.6536	0.7792
I:Fu vs. III:Fu	0.02540	No	0.6281	0.7352
I:Fu vs. III:E	-0.01564	No	0.6735	0.8337
I:Fu vs. IV:C	0.03500	No	0.5886	0.6590
I:Fu vs. IV:Fe	0.03274	No	0.5968	0.6797
I:Fu vs. IV:Fu	0.02842	No	0.6190	0.7200
I:Fu vs. IV:E	0.02269	No	0.6536	0.7748
I:Fu vs. V:C	-0.1337	No	0.1669	0.0922
I:Fu vs. V:Fe	-0.3843	Yes	<0.0001	<0.0001
I:Fu vs. V:Fu	-0.05377	No	0.5108	0.5125
I:Fu vs. V:E	-0.04191	No	0.5513	0.5972
I:E vs. II:C	-0.1207	No	0.2739	0.1633
I:E vs. II:Fe	-0.2635	Yes	0.0071	0.0024
I:E vs. II:Fu	-0.01988	No	0.6660	0.8183

I:E vs. II:E	-0.2041	Yes	0.0421	0.0186
I:E vs. III:C	0.06205	No	0.4618	0.4068
I:E vs. III:Fe	0.05381	No	0.4893	0.4702
I:E vs. III:Fu	0.1001	No	0.2892	0.1829
I:E vs. III:E	0.05905	No	0.4703	0.4281
I:E vs. IV:C	0.1097	No	0.2767	0.1670
I:E vs. IV:Fe	0.1074	No	0.2813	0.1759
I:E vs. IV:Fu	0.1031	No	0.2996	0.1939
I:E vs. IV:E	0.09738	No	0.3117	0.2198
I:E vs. V:C	-0.05902	No	0.4834	0.4569
I:E vs. V:Fe	-0.3096	Yes	0.0007	0.0002
I:E vs. V:Fu	0.02092	No	0.6644	0.7988
I:E vs. V:E	0.03278	No	0.5968	0.6794
II:C vs. II:Fe	-0.1428	No	0.1250	0.0654
II:C vs. II:Fu	0.1009	No	0.2996	0.1929
II:C vs. II:E	-0.08335	No	0.3726	0.2817
II:C vs. III:C	0.1828	Yes	0.0120	0.0044
II:C vs. III:Fe	0.1745	Yes	0.0157	0.0063
II:C vs. III:Fu	0.2208	Yes	0.0024	0.0006
II:C vs. III:E	0.1798	Yes	0.0129	0.0049
II:C vs. IV:C	0.2304	Yes	0.0033	0.0009
II:C vs. IV:Fe	0.2282	Yes	0.0035	0.0010
II:C vs. IV:Fu	0.2239	Yes	0.0042	0.0013
II:C vs. IV:E	0.2181	Yes	0.0053	0.0017
II:C vs. V:C	0.06172	No	0.4420	0.3728
II:C vs. V:Fe	-0.1889	Yes	0.0217	0.0093
II:C vs. V:Fu	0.1417	No	0.1013	0.0508
II:C vs. V:E	0.1535	No	0.0579	0.0269
II:Fe vs. II:Fu	0.2437	Yes	0.0053	0.0017
II:Fe vs. II:E	0.05946	No	0.4765	0.4424
II:Fe vs. III:C	0.3256	Yes	<0.0001	<0.0001
II:Fe vs. III:Fe	0.3174	Yes	<0.0001	<0.0001
II:Fe vs. III:Fu	0.3636	Yes	<0.0001	<0.0001
II:Fe vs. III:E	0.3226	Yes	<0.0001	<0.0001
II:Fe vs. IV:C	0.3732	Yes	<0.0001	<0.0001
II:Fe vs. IV:Fe	0.3710	Yes	<0.0001	<0.0001
II:Fe vs. IV:Fu	0.3667	Yes	<0.0001	<0.0001
II:Fe vs. IV:E	0.3609	Yes	<0.0001	<0.0001
II:Fe vs. V:C	0.2045	Yes	0.0091	0.0033
II:Fe vs. V:Fe	-0.04607	No	0.5126	0.5246
II:Fe vs. V:Fu	0.2845	Yes	0.0004	<0.0001
II:Fe vs. V:E	0.2963	Yes	0.0001	<0.0001
II:Fu vs. II:E	-0.1842	Yes	0.0404	0.0176
II:Fu vs. III:C	0.08193	No	0.3002	0.2008
II:Fu vs. III:Fe	0.07369	No	0.3401	0.2473
II:Fu vs. III:Fu	0.1200	No	0.1215	0.0627
II:Fu vs. III:E	0.07893	No	0.3085	0.2153
II:Fu vs. IV:C	0.1296	No	0.1211	0.0616
II:Fu vs. IV:Fe	0.1273	No	0.1250	0.0663
II:Fu vs. IV:Fu	0.1230	No	0.1413	0.0760
II:Fu vs. IV:E	0.1173	No	0.1663	0.0907
II:Fu vs. V:C	-0.03913	No	0.5436	0.5719
II:Fu vs. V:Fe	-0.2897	Yes	0.0003	<0.0001

II:Fu vs. V:Fu	0.04080	No	0.5436	0.5731
II:Fu vs. V:E	0.05266	No	0.4765	0.4469
II:E vs. III:C	0.2661	Yes	0.0002	<0.0001
II:E vs. III:Fe	0.2579	Yes	0.0003	<0.0001
II:E vs. III:Fu	0.3042	Yes	<0.0001	<0.0001
II:E vs. III:E	0.2631	Yes	0.0002	<0.0001
II:E vs. IV:C	0.3138	Yes	<0.0001	<0.0001
II:E vs. IV:Fe	0.3115	Yes	<0.0001	<0.0001
II:E vs. IV:Fu	0.3072	Yes	<0.0001	<0.0001
II:E vs. IV:E	0.3015	Yes	<0.0001	<0.0001
II:E vs. V:C	0.1451	No	0.0749	0.0365
II:E vs. V:Fe	-0.1055	No	0.2530	0.1453
II:E vs. V:Fu	0.2250	Yes	0.0059	0.0020
II:E vs. V:E	0.2369	Yes	0.0024	0.0007
III:C vs. III:Fe	-0.008240	No	0.6775	0.8592
III:C vs. III:Fu	0.03804	No	0.4686	0.4224
III:C vs. III:E	-0.003002	No	0.7052	0.9485
III:C vs. IV:C	0.04764	No	0.4423	0.3762
III:C vs. IV:Fe	0.04538	No	0.4616	0.3993
III:C vs. IV:Fu	0.04107	No	0.4765	0.4456
III:C vs. IV:E	0.03533	No	0.5108	0.5116
III:C vs. V:C	-0.1211	No	0.0542	0.0248
III:C vs. V:Fe	-0.3717	Yes	<0.0001	<0.0001
III:C vs. V:Fu	-0.04113	No	0.4898	0.4772
III:C vs. V:E	-0.02927	No	0.5456	0.5866
III:Fe vs. III:Fu	0.04628	No	0.4020	0.3244
III:Fe vs. III:E	0.005238	No	0.6935	0.9093
III:Fe vs. IV:C	0.05588	No	0.3838	0.2957
III:Fe vs. IV:Fe	0.05362	No	0.3948	0.3157
III:Fe vs. IV:Fu	0.04930	No	0.4298	0.3562
III:Fe vs. IV:E	0.04357	No	0.4639	0.4149
III:Fe vs. V:C	-0.1128	No	0.0731	0.0351
III:Fe vs. V:Fe	-0.3634	Yes	<0.0001	<0.0001
III:Fe vs. V:Fu	-0.03289	No	0.5436	0.5672
III:Fe vs. V:E	-0.02103	No	0.6040	0.6938
III:Fu vs. III:E	-0.04104	No	0.4455	0.3822
III:Fu vs. IV:C	0.009605	No	0.6775	0.8595
III:Fu vs. IV:Fe	0.007340	No	0.6934	0.8924
III:Fu vs. IV:Fu	0.003025	No	0.7063	0.9555
III:Fu vs. IV:E	-0.002708	No	0.7063	0.9602
III:Fu vs. V:C	-0.1591	Yes	0.0096	0.0035
III:Fu vs. V:Fe	-0.4097	Yes	<0.0001	<0.0001
III:Fu vs. V:Fu	-0.07917	No	0.2813	0.1745
III:Fu vs. V:E	-0.06731	No	0.3085	0.2151
III:E vs. IV:C	0.05065	No	0.4216	0.3433
III:E vs. IV:Fe	0.04838	No	0.4369	0.3653
III:E vs. IV:Fu	0.04407	No	0.4618	0.4096
III:E vs. IV:E	0.03833	No	0.4893	0.4731
III:E vs. V:C	-0.1181	No	0.0581	0.0275
III:E vs. V:Fe	-0.3687	Yes	<0.0001	<0.0001
III:E vs. V:Fu	-0.03813	No	0.5108	0.5071
III:E vs. V:E	-0.02627	No	0.5637	0.6229
IV:C vs. IV:Fe	-0.002265	No	0.7096	0.9699

IV:C vs. IV:Fu	-0.006580	No	0.6935	0.9126
IV:C vs. IV:E	-0.01231	No	0.6735	0.8373
IV:C vs. V:C	-0.1687	Yes	0.0131	0.0050
IV:C vs. V:Fe	-0.4193	Yes	<0.0001	<0.0001
IV:C vs. V:Fu	-0.08878	No	0.2739	0.1630
IV:C vs. V:E	-0.07692	No	0.3002	0.1998
IV:Fe vs. IV:Fu	-0.004315	No	0.7047	0.9426
IV:Fe vs. IV:E	-0.01005	No	0.6775	0.8669
IV:Fe vs. V:C	-0.1664	Yes	0.0144	0.0057
IV:Fe vs. V:Fe	-0.4171	Yes	<0.0001	<0.0001
IV:Fe vs. V:Fu	-0.08651	No	0.2813	0.1740
IV:Fe vs. V:E	-0.07465	No	0.3085	0.2134
IV:Fu vs. IV:E	-0.005733	No	0.6982	0.9238
IV:Fu vs. V:C	-0.1621	Yes	0.0173	0.0070
IV:Fu vs. V:Fe	-0.4127	Yes	<0.0001	<0.0001
IV:Fu vs. V:Fu	-0.08220	No	0.3002	0.1965
IV:Fu vs. V:E	-0.07034	No	0.3348	0.2410
IV:E vs. V:C	-0.1564	Yes	0.0217	0.0093
IV:E vs. V:Fe	-0.4070	Yes	<0.0001	<0.0001
IV:E vs. V:Fu	-0.07646	No	0.3221	0.2295
IV:E vs. V:E	-0.06460	No	0.3726	0.2814
V:C vs. V:Fe	-0.2506	Yes	0.0004	<0.0001
V:C vs. V:Fu	0.07994	No	0.3085	0.2090
V:C vs. V:E	0.09180	No	0.2224	0.1261
V:Fe vs. V:Fu	0.3305	Yes	<0.0001	<0.0001
V:Fe vs. V:E	0.3424	Yes	<0.0001	<0.0001
V:Fu vs. V:E	0.01186	No	0.6775	0.8520

[1] Proteins are grouped as follows: I – RPO (subunits of DNA-directed RNA polymerase); II – ATPase (subunits of F_0F_1-type ATP synthase); III – ETC (subunits of type I NADH-dehydrogenase and membrane-bound succinate dehydrogenase/fumarate reductase); IV – pili (proteins of pili assembly encoded in the 'pilin-cytochrome' cluster); V – multihemes (multiheme cytochromes which are predicted to be secreted or cell surface-associated proteins). Refer to the text for details.
[2] Electron acceptors are given as follows: C – ferric citrate; E – anode (electrogenic cells); Fe – ferrihydrite; Fu – fumarate.

Table S4. Pairwise comparisons of representation of each of multiheme cytochromes[1] at each of cultured conditions tested (four different electron acceptors).

Pairs (representation of each cytochrome across four different electron acceptors[2])	Mean Diff.	Discovery?	q value	Individual P Values
Ga0395992_01_187284_188528				
C vs. Fe	-0.02639	No	>0.9999	0.6895
C vs. Fu	-0.003339	No	>0.9999	0.9597
C vs. E	-0.007139	No	>0.9999	0.9139
Fe vs. Fu	0.02305	No	>0.9999	0.7270
Fe vs. E	0.01926	No	>0.9999	0.7706
Fu vs. E	-0.003799	No	>0.9999	0.9541
Ga0395992_01_217646_219709				
C vs. Fe	0.02219	No	>0.9999	0.7369
C vs. Fu	0.02219	No	>0.9999	0.7369

C vs. E	0.02134	No	>0.9999	0.7466
Fe vs. Fu	-1.110e-016	No	>0.9999	>0.9999
Fe vs. E	-0.0008433	No	>0.9999	0.9898
Fu vs. E	-0.0008433	No	>0.9999	0.9898
Ga0395992_01_222075_224618				
C vs. Fe	0.001526	No	>0.9999	0.9816
C vs. Fu	0.001526	No	>0.9999	0.9816
C vs. E	0.001118	No	>0.9999	0.9865
Fe vs. Fu	-2.220e-016	No	>0.9999	>0.9999
Fe vs. E	-0.0004085	No	>0.9999	0.9951
Fu vs. E	-0.0004085	No	>0.9999	0.9951
Ga0395992_02_29558_30178				
C vs. Fe	0.006299	No	>0.9999	0.9240
C vs. Fu	0.01925	No	>0.9999	0.7707
C vs. E	0.01948	No	>0.9999	0.7680
Fe vs. Fu	0.01295	No	>0.9999	0.8445
Fe vs. E	0.01318	No	>0.9999	0.8417
Fu vs. E	0.0002337	No	>0.9999	0.9972
Ga0395992_02_30996_31874				
C vs. Fe	-0.007666	No	>0.9999	0.9076
C vs. Fu	0.003865	No	>0.9999	0.9533
C vs. E	-0.006594	No	>0.9999	0.9205
Fe vs. Fu	0.01153	No	>0.9999	0.8614
Fe vs. E	0.001072	No	>0.9999	0.9870
Fu vs. E	-0.01046	No	>0.9999	0.8741
OmhA				
C vs. Fe	-2.031	Yes	<0.0001	<0.0001
C vs. Fu	0.1838	Yes	0.0014	0.0065
C vs. E	0.2800	Yes	<0.0001	<0.0001
Fe vs. Fu	2.215	Yes	<0.0001	<0.0001
Fe vs. E	2.311	Yes	<0.0001	<0.0001
Fu vs. E	0.09620	Yes	0.0259	0.1478
Ga0395992_02_135863_137242				
C vs. Fe	0.003549	No	>0.9999	0.9571
C vs. Fu	0.009265	No	>0.9999	0.8884
C vs. E	-0.009986	No	>0.9999	0.8798
Fe vs. Fu	0.005716	No	>0.9999	0.9310
Fe vs. E	-0.01354	No	>0.9999	0.8376
Fu vs. E	-0.01925	No	>0.9999	0.7707
SmhA				
C vs. Fe	-0.1738	Yes	0.0209	0.0099
C vs. Fu	-0.1038	No	0.1246	0.1187
C vs. E	-0.2275	Yes	0.0037	0.0009
Fe vs. Fu	0.06999	No	0.2443	0.2908
Fe vs. E	-0.05366	No	0.2921	0.4174
Fu vs. E	-0.1236	No	0.0895	0.0639
SmhC				
C vs. Fe	0.1596	Yes	0.0037	0.0176

C vs. Fu	0.4667	Yes	<0.0001	<0.0001
C vs. E	0.4403	Yes	<0.0001	<0.0001
Fe vs. Fu	0.3071	Yes	<0.0001	<0.0001
Fe vs. E	0.2807	Yes	<0.0001	<0.0001
Fu vs. E	-0.02646	No	0.1205	0.6888
SmhB				
C vs. Fe	0.4228	Yes	<0.0001	<0.0001
C vs. Fu	0.4219	Yes	<0.0001	<0.0001
C vs. E	0.4069	Yes	<0.0001	<0.0001
Fe vs. Fu	-0.0009423	No	0.5190	0.9886
Fe vs. E	-0.01597	No	0.5166	0.8089
Fu vs. E	-0.01503	No	0.5166	0.8199

[1] Refer to the text for detailed description of the cytochromes, highlighted yellow are the data for four predominant cytochromes presumed to determine EET activity in *C. ferrireducens*.
[2] Electron acceptors are given as follows: C – ferric citrate; E – anode (electrogenic cells); Fe – ferrihydrite; Fu – fumarate.

Supplementary methods

Inoculation techniques. The culture was maintained on the medium with ferrihydrite (90 mM initial insoluble Fe(III) content) in Hungate tubes. To get the biomass for proteomic studies the cultures were transferred to 100 mL bottles sealed with rubber stoppers, for OmhA protein isolation the cultures were further transferred to 10 L bottles. All the transfers were made anaerobically with syringes and needles, 1% inoculum volume was used throughout. For proteomic profiling across different electron acceptors the cultures were three times subsequently transferred from ferrihydrite medium to the media with corresponding electron acceptors (20 mM fumarate or ferric citrate) and third transfers in 100 mL bottles were used for biomass harvesting. The 10 L bottles for mass cultivation initially contained the medium without electron acceptors and were inoculated with ferrihydrite-grown cultures with residual magnetite. Thus, the total Fe content of the medium after inoculation comprised ca. 600 µM (200 µM Fe(II) and 400 µM Fe(III)). BESs were also inoculated with ferrihydrite-grown cells but after preliminary separation of the bulk of magnetite with a neodymium hand magnet attached to the Hungate tube.
In all the cultivation vessels, except BESs, 70% of the vessel volume was filled with liquid medium, and 30% - with pure CO_2 gas. In the BESs, anodic chambers were completely filled with the liquid medium lacking any electron acceptors.

BESs experiments. Dual chamber BESs with Nafion 242 proton-exchange membranes (DuPont) were used: one on MFC mode, and another – on potentiostatic mode at +10 mV vs SHE. All the BES parts (except membranes and reference electrodes) were autoclaved at 121ºC for 20 min and further assembled and filled with pre-sterilized culture medium aseptically in an anaerobic glove box (98% N_2, 2% H_2), Nafion membrane and Ag/AgCl reference electrode (+200 mV vs SHE, RE-5B, BASi, IN, USA) were UV-irradiated for 20 min before BESs assembling. In MFC reactors, graphite felt electrodes (GF-S6-06; Electrolytica) bonded to platinum wires (0.3 mm; Alfa-Aesar) were used, while potentiostatic mode reactors were assembled with stainless steel 240 mesh electrodes bonded to 0.4 mm titanium wires (both from Joyetech, China) to simplify shearing of biofilms and extracellular proteins from anodes. The culture medium lacking external electron acceptors was used in anodic chambers of both BESs types. Cathodic chambers in potentiostatic mode BESs were filled with the same sterile culture medium, which was aerated during the incubation period via syringe needles placed through rubber stoppers to avoid catholyte evaporation at 65ºC. In MFCs, cathodic chambers were filled with potassium ferricyanide solution. MFCs were incubated for 3 days with open circuit to

pregrow biofilms on graphite surface, and were further operated with a 200 Ω resistor load. Before closing the circuit, samples of the pregrown *C. ferrireducens* biofilms were collected anaerobically, stained with SYBR Green DNA stain, and imaged using a Leica confocal laser microscope. The potentiostat mode BESs were turned on right after inoculation with anodes poised at to -60 mV vs SHE. Anode potential was controlled using Ag/AgCl reference electrodes with an IPC-micro potentiostats (Cronas, Russia). Potentiostatic BES systems were operated constantly for 9 days with phase-contrast microscopic control of cell growth until appearance of lysing planktonic cells in the anodic chamber that corresponded to the formation of visible biofilms on the anodic surface. These biofilms were used to obtain biomass of electrogenic cells for proteomic studies. The biofilms were sheared from stainless steel anodes by their rigorous shaking in the culture broth, and the cells were separated by centrifugation at 16000 g 15 min.

Samples preparation from SDS-PAGE gels for MS analyzes. Protein bands of interest were excised from gels and dehydrated using acetonitrile followed by vacuum centrifugation. Dried gel pieces were reduced with 10 mM dithiothreitol and alkylated with 55 mM iodoacetamide. Gel pieces were then washed alternately with 25 mM ammonium bicarbonate followed by acetonitrile. This was repeated, and the gel pieces were dried by vacuum centrifugation. Samples were digested with trypsin overnight at 37°C. Peptide mixtures of tryptic digests were separated by liquid chromatography with a gradient from 92% solution A (0.1% formic acid (FA) in water) and 8% solution B (0.1% FA in acetonitrile) to 77% A and 33% B, in 44 min at 300 nL min^{-1}, using a 0.25 x 75 mm, 1.7 µm, ethylene bridged hybrid (BEH) C18 analytical column (Waters). Peptides were selected for fragmentation automatically by data-dependent analysis.

Modifications of shotgun proteomic analysis technique. Briefly, sodium deoxycholate (SDC) lysis, reduction and alkylation buffer (pH 8.5) containing 100 mM TRIS, 1% (w/v) SDC, 10 mM TRIS(2-carboxyethyl)phosphine (TCEP) and 40 mM 2-chloroacetamide was added to a cell sample. The sample was sonicated and boiled for 10 min, protein concentration in the sample was routinely determined by Bradford assay and equal volume of trypsin solution in 100 mM TRIS, pH 8.5, was added in a 1:100 (w/w) ratio. After overnight digestion at 37°C, peptides were acidified by 1% trifluoroacetic acid (TFA) for styrenedivinylbenzene-reverse phase sulfonate (SDB-RPS)-binding, and 20 µg was loaded on two 14-gauge StageTip plugs. Equal volume of ethyl acetate was added, and the StageTips were centrifuged at 300 g. After washing the StageTips with a 100 µl of 1% TFA/ethyl acetate mixture and 100 µl of 0.2% TFA, peptides were eluted by 60 µl acetonitrile/ammonia (80/5 %) mixture. The collected material was vacuum-dried and stored at -80°C. Before analysis, peptides were dissolved in 2% acetonitrile/0.1% TFA buffer) and sonicated for 2 min. Dissolved peptides were further analyzed using the HPLC system configured in a trap-elute mode. Approximately 1 µg of tryptic peptide digests were loaded on an Acclaim PepMap 100 (100 µm x 2 cm) trap column and separated on an Acclaim PepMap 100 (75 µm x 50 cm) column (both from Thermo Fisher Scientific). Peptides were loaded in solvent A (0.2% FA) and eluted at a flow rate of 350 nL min^{-1} with a multistep linear gradient of solvent B (0.1% FA, 80% acetonitrile): 4 - 6% B for 5 min; 6 – 28% B for 91 min; 28 – 45% B for 20 min; 45 – 99% B for 4 min; 99% B for 7 min; 99 – 4% B for 1 min. After each gradient, the column was washed with 96% buffer B for 9 min. Column temperature was kept at 40°C. Peptides were analyzed on a mass spectrometer with one full scan (375–1,400 *m/z*, *R* = 60,000 at 200 *m/z*) at a target of 3e6 ions and max ion fill time 30 ms, followed by up to 15 data-dependent MS/MS scans with higher-energy collisional dissociation (HCD) (target 1e5 ions, max ion fill time 50 ms, isolation window 1.2 m/z, normalized collision energy (NCE) 28%, underfill ratio 2%), detected in the Orbitrap (*R* = 15,000 at fixed first mass 100 *m/z*). Other settings: charge exclusion - unassigned, 1, >6; peptide match – preferred; exclude isotopes – on; dynamic exclusion - 30 s was enabled.

For label-free protein quantification by MaxQuant software version 1.5.6.5 was used with *Carboxydothermus ferrireducens* amino acid FASTA database from IMG JGI and a common contaminants database by the Andromeda search engine, with cysteine carbamidomethylation as a fixed modification and protein N-terminal acetylation and methionine oxidations as variable

modifications. The false discovery rate (FDR) was set to 0.01 for both proteins and peptides with a minimum length of seven amino acids. Peptide identification was performed with an allowed initial precursor mass deviation up to 20 ppm and an allowed fragment mass deviation of 20 ppm. Downstream bioinformatics analysis was performed with Perseus.1.5.5.1. For Student's t-test, missing values were imputed with a width of 0.3 and a downshift of 1.8 over the total matrix. Two sample tests were performed in Perseus with s0 set to 0. Quantification was performed with a minimum ratio count of 1.
